# A funnel approach to enable analyses of epitope-specific human CD4 T cells specific for influenza and SARS-CoV-2

**DOI:** 10.1128/mbio.00449-26

**Published:** 2026-05-19

**Authors:** K. A. Richards, R. C. Mettelman, K. Trombly, M. Eismann, E. K. Allen, Z. B. Scott, A. Joachimiak, S. Schultz-Cherry, F. A. Chaves, P. G. Thomas, A. J. Sant

**Affiliations:** 1 David H. Smith Center for Vaccine Biology and Immunology, Department of Microbiology and Immunology, University of Rochester 6927, Rochester, New York, USA; 2 Department of Host–Microbe Interactions, St. Jude Children's Research Hospital 5417, Memphis, Tennessee, USA; 3 Center for Structural Biology of Infectious Diseases, Consortium for Advanced Science and Engineering, University of Chicago 2462, Chicago, Illinois, USA; 4 Structural Biology Center, X-ray Science Division, Argonne National Laboratory 1291, Lemont, Illinois, USA; 5 Department of Biochemistry and Molecular Biology, University of Chicago 2462, Chicago, Illinois, USA; Monash University 445705, Clayton, Victoria, Australia

**Keywords:** influenza, SARS-CoV-2, CD4 T cell, epitopes

## Abstract

Protection against pathogens relies heavily on the adaptive immune response, whose key regulators are CD4 T cells. CD4 T cells, notable for their complex repertoire and functional potential, can most easily be dissected by identifying, quantifying, characterizing, and isolating epitope-specific cells. In the study reported here, we present a systematic and unbiased strategy that has enabled the identification of highly immunogenic peptide epitopes derived from influenza virus and SARS-CoV-2, presented by human HLA-DR proteins. Coupling the use of HLA-DR transgenic mice with infection and vaccination and highly sensitive epitope-specific cytokine ELISpot assays, we have narrowed the potential epitopes from 450 to 600 peptides to 5–15 peptides for each allele by an iterative process of elimination and selection, which we have termed a funnel approach. These epitopes have been validated in HLA-DR-typed human CD4 T cells directly *ex vivo* and enabled the derivation and implementation of HLA-DR peptide tetramers. Tetramer staining of human PBMCs enriched for CD4 T memory populations from healthy adult subjects, highlighted this approach as a sensitive and specific method for identifying novel epitopes, and subsequent CD4 T-cell responses to human viral infections.

**IMPORTANCE:**

Tracking single epitope-specific CD4 T cells enables sophisticated analyses of the human response to infectious pathogens, vaccines, and probing the human CD4 T-cell immune memory compartment. The studies presented here provide an unbiased strategy for accomplishing this goal and provide a verified compilation of candidate HLA-DR-restricted CD4 T-cell peptide epitopes for future studies by researchers in the field of human immunology.

## INTRODUCTION

Identification of single peptide epitopes recognized by human T cells offers tremendous advantages for understanding human cellular immunity. These epitope-specific T cells vary in their history, number of boosts, or the original site of priming, all of which can impact their fate and effector functions. In cellular immunology, a notable advance is the identification of single peptides that are “restricted” (e.g., bound and presented) by a known HLA molecule. Such progress can enable the derivation of peptide:HLA multimers for the isolation of epitope-specific T cells. Tetramer-based isolation of T cells can be followed by the evaluation of T-cell receptor repertoire, transcriptomics, partitioning of T cells into memory subsets, or the expression of transcription factors associated with specific functions. These analyses can be performed without the need to stimulate the T cell populations, which may alter gene expression patterns (for reviews, see references 1–3). Epitope discovery and tracking of T cells with single peptide specificity can also provide insights into the factors that shape immunodominance in T-cell responses, the functionality of the polyclonal responding T cells, and their persistence in the face of antigenic drift (4–9). Epitope discovery also enables the derivation of peptide-based vaccines comprised of conserved peptide epitopes (10–12).

To identify peptides presented by particular HLA molecules, some investigators have utilized a strategy termed tetramer-guided epitope mapping (TGEM) (13), which involves the derivation of tetramers with candidate peptides, pooling them, and identifying positive tetramers, and thus the peptide:HLA complexes through a sequential process of de-convolution. Also, advances in bioinformatics have been useful tools for epitope discovery, particularly for HLA-class I and class II molecules with well-defined “motifs” that correlate with peptide acquisition (14–17). A labor-intensive and technically complex strategy for epitope discovery involves the isolation of HLA-class II:peptide complex molecules from transfected cells expressing a single MHC-class I or -class II molecule, either infected or pulsed with antigens, followed by elution and sequencing of the eluted, HLA-bound peptides, yielding the “immunopeptidome” of that pair of HLA-class II molecules and pathogens (18–24). This approach ensures that the identified peptides have stable interactions with their presenting HLA molecules, a factor associated with immunodominance (25, 26). This method has also been particularly useful for the identification of peptides presented by rare HLA molecules whose peptide binding “motifs” are poorly defined (see (27)). Finally, a procedure termed “reverse epitope discovery” using cloned T-cell receptors can be employed for the identification of peptide:HLA complexes (28). The approach we describe here for influenza and SARS-CoV-2, which provides a blueprint for any human pathogen of interest, is completely unbiased, starting with direct *ex vivo* T-cell sampling after infection or vaccination of HLA-transgenic animals. HLA-DR transgenic mice in combination with cytokine ELISpot assays are used in an iterative, completely empirical assay to identify single viral peptide epitopes that are identified in the primary response, thus reflecting natural immunodominance selection. We follow this epitope discovery stage by testing immunogenicity in HLA-DR-typed human PBMC directly *ex vivo* with no *in vitro* antigen-stimulated cell expansion as other studies have employed (13, 29–31). Finally, after completion of these epitope identification and verification studies, we have derived HLA-class II multimers on HLA-typed human samples to dissect the phenotype of circulating CD4 T-cell memory cells specific for several viral pathogen-derived peptides.

## RESULTS

The goals for this study were to evaluate the success of a completely empirical approach to identify single pathogen-derived epitopes recognized by CD4 T cells that are presented by specific HLA-DR proteins, as a model that can be implemented for any pathogen of interest. The presented strategy is used to narrow the repertoire (e.g., create a “funnel”) of potential epitopes in multiple proteins from influenza and SARS-CoV-2 from 800 to 1,000 peptides to less than 20 single HLA-DR-restricted peptide epitopes recognized by CD4 T cells in HLA-DR-typed humans. A key strategy for these studies was to use humanized mice, expressing single HLA-DR molecules, to identify pathogen-derived peptides as the first step for ultimate validation in human CD4 T cells. The approach focused on four commonly expressed HLA-DR alleles (32): HLA-DR1 (Β1*01:01), HLA-DR4 (Β1*04:01), HLA-DR15 (Β1*15:01), and HLA-DR3 (Β1*03:01). T-cell epitope discovery was initiated by acquisition and utilization of the four HLA-DR transgenic mice that express HLA-DR alleles that are highly represented in the US populations (32), which collectively have more than 40%–50% population coverage (32).

**Fig 1:**
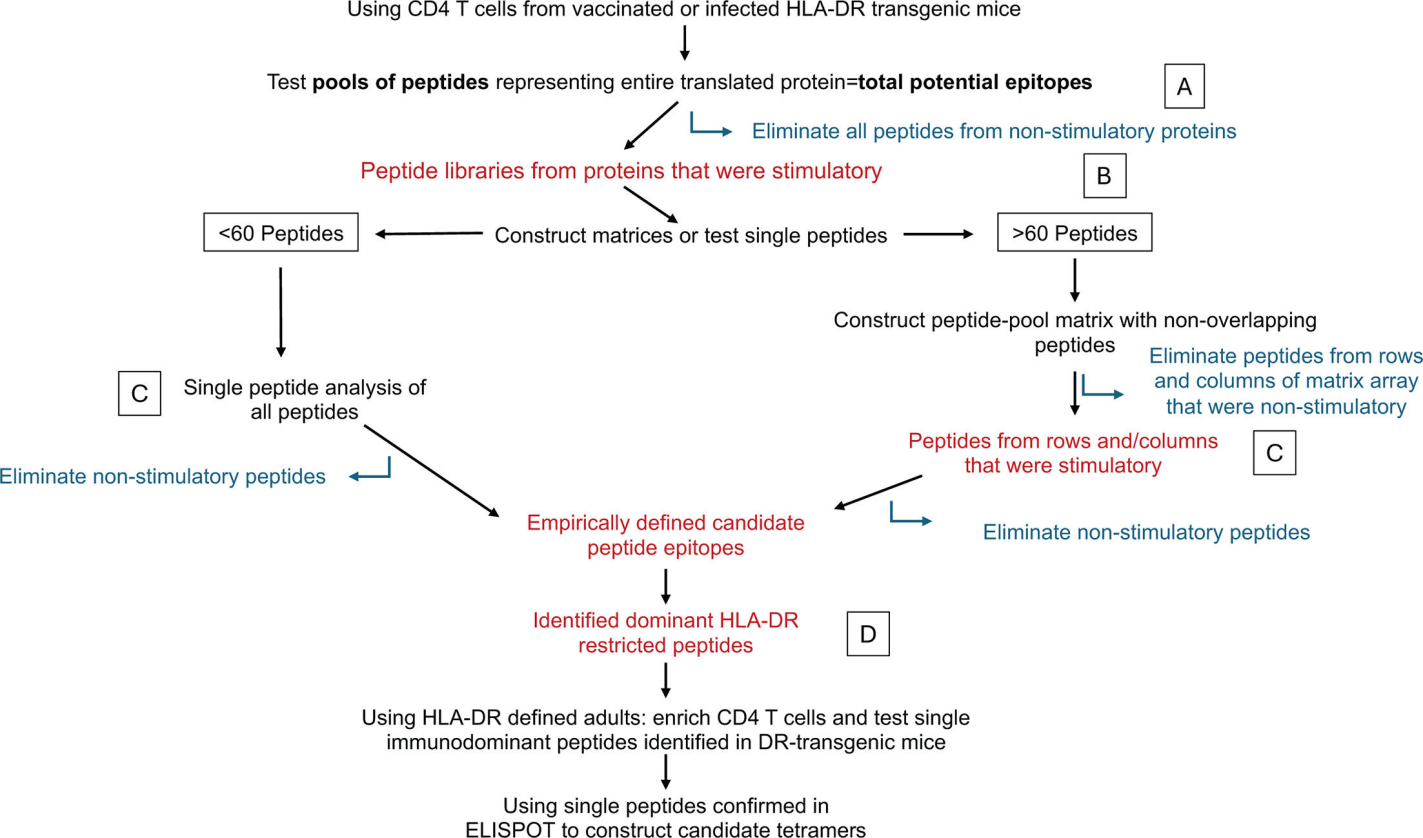
Schematic of the iterative approach for epitope discovery in HLA-DR transgenic mice and analyses directly *ex vivo* in human CD4 T cells. Shown is the experimental path that was used here and that can be applied to any pathogen or antigen of interest for the identification of dominant epitopes directly *ex vivo*. The sequential iterative process narrows the candidates, with non-stimulatory pools or individual peptides identified in blue and the positive pools or peptides identified in red font. The boxed letters A–D represent the “slices” of the funnels shown in Fig. 9.

**Fig 2:**
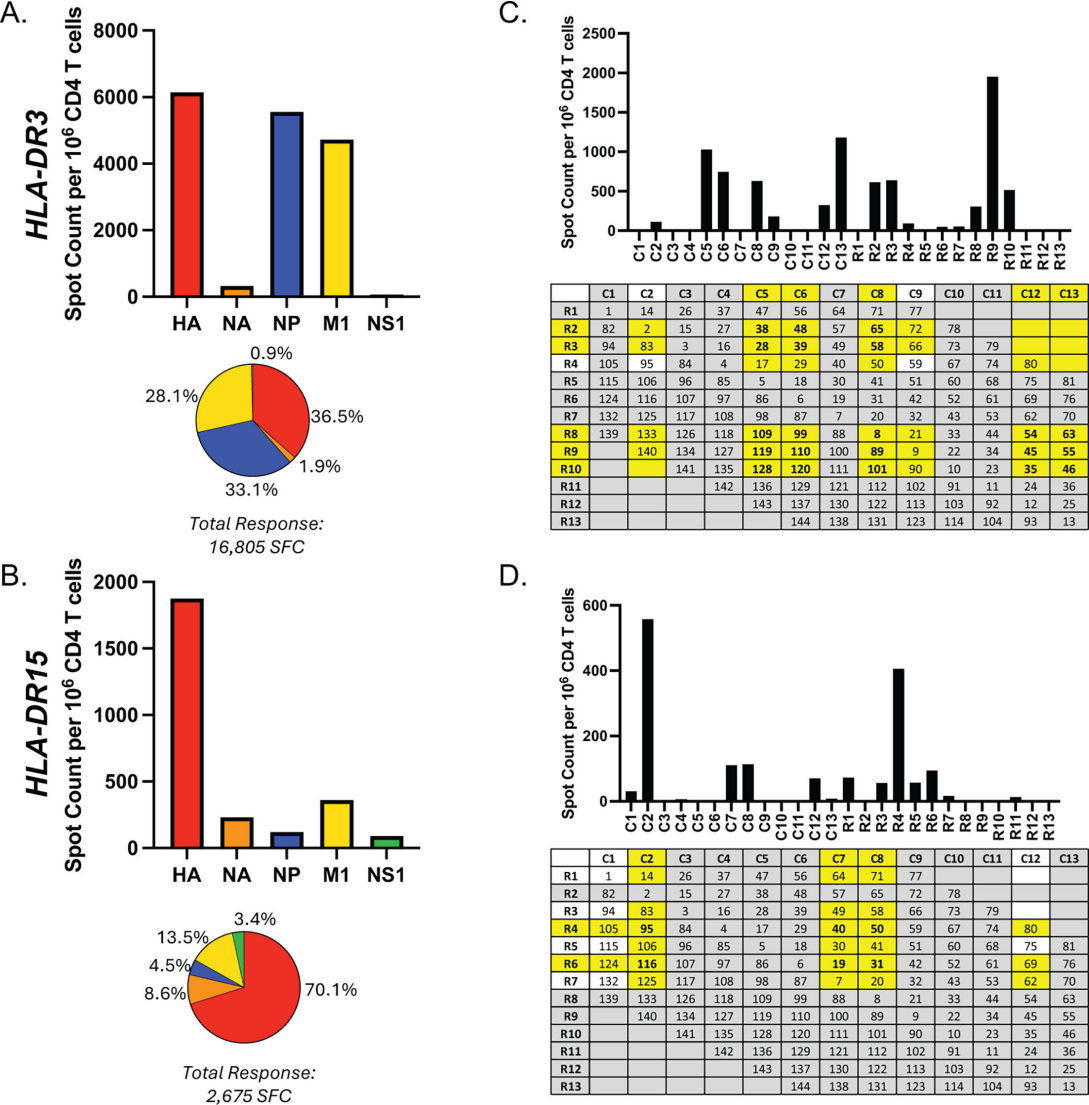
The experimental path to identifying influenza B-specific CD4 T-cell epitopes. HLA-DRB1*03:01 and HLA-DRB1*15:01 mice were infected with influenza B/Brisbane/60/08 virus. CD4 T cells from the draining lymph node were restimulated with pools of peptides encompassing the entire translated regions of HA (red), NA (orange), NP (blue), M1 (yellow), and NS1 (green). Shown in panels **A** and **B** are the frequencies of IFN-γ producing cells per million CD4 T cells as bar graphs, with the percent of the response to each protein shown as a pie chart below and the total number of spot-forming cells (SFC per million) beneath each pie. Panels **C** (HLA-DRB1*03:01, “HLA-DR3”) and **D** (HLA-DRB1*15:01, “HLA-DR15”) illustrate the responses to the HA-B peptide matrix, where peptides are grouped into pools denoted as rows (“R”) and columns (“C”). The peptide composition of each pool is indicated with each peptide represented by single peptide numbers 1–144. Pools were considered positive with a response at least threefold over background. Non-stimulatory pools are indicated in gray, stimulatory pools are indicated in yellow, and pools in white were stimulatory but weak. Peptides at the intersection points are potentially the immunodominant epitopes, which were further tested in single peptide analyses.

**Fig 3:**
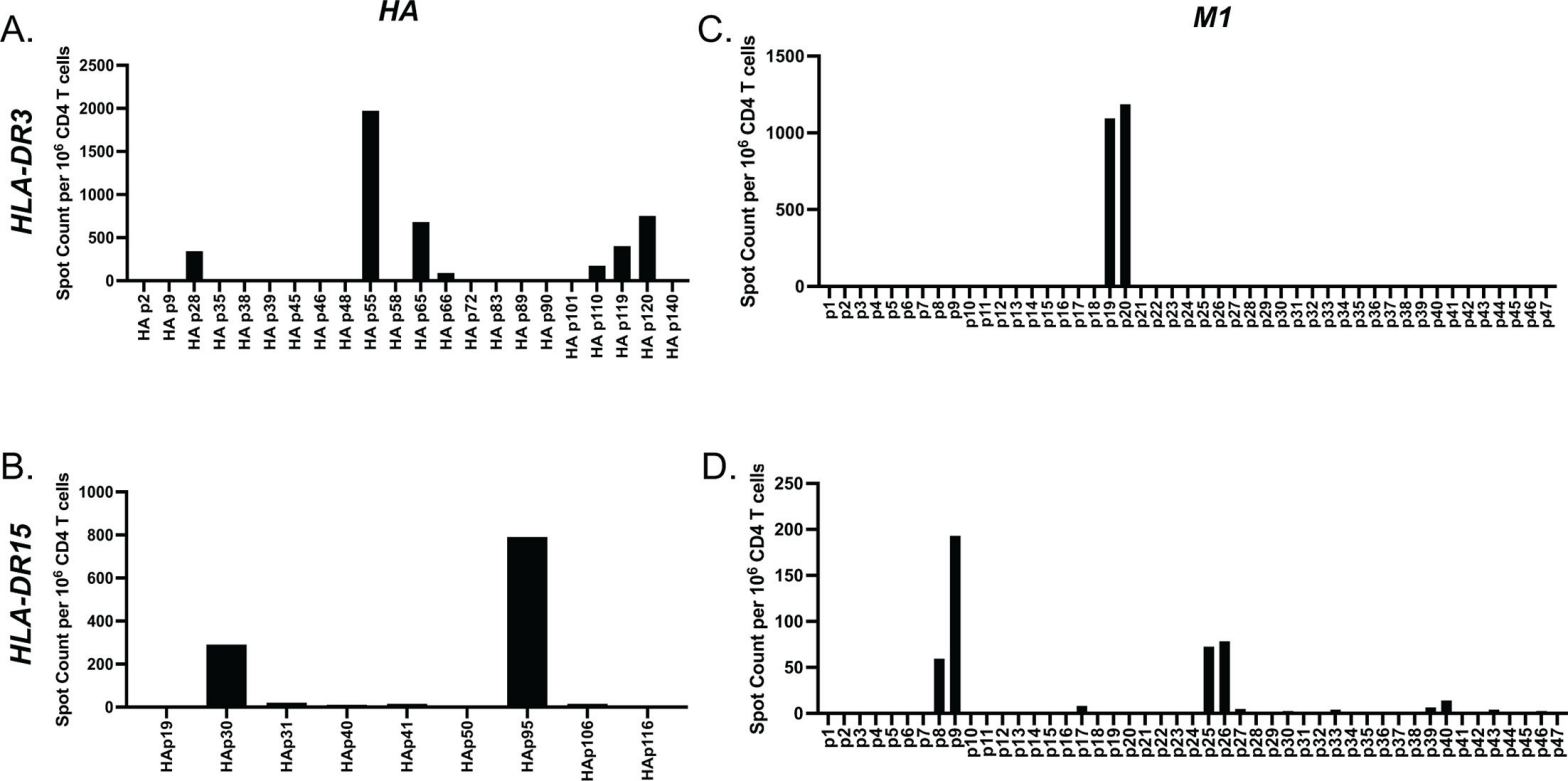
Single peptide analysis of the reactivity to HA and M1 proteins of influenza B-specific CD4 T-cell epitopes after infection. Shown here are representative plots of single peptide analyses for identification of CD4 T-cell peptide epitopes in HA (left, **A and B**) and M1 (right, **C and D**) proteins of influenza B/Brisbane/60/08 after infection in HLA-DR3 (top, **A and C**) or HLA-DR15 (bottom, **B and D**). The peptides selected for analysis for HA-B are based on the stimulatory peptides and peptides eliminated in the matrices shown in Fig. 2. All M1 peptides were tested individually. Shown are the responses of splenic CD4 T cells, with the bar graphs representing the average frequency of IFN-γ-producing cells per million, with background subtracted.

**Fig 4:**
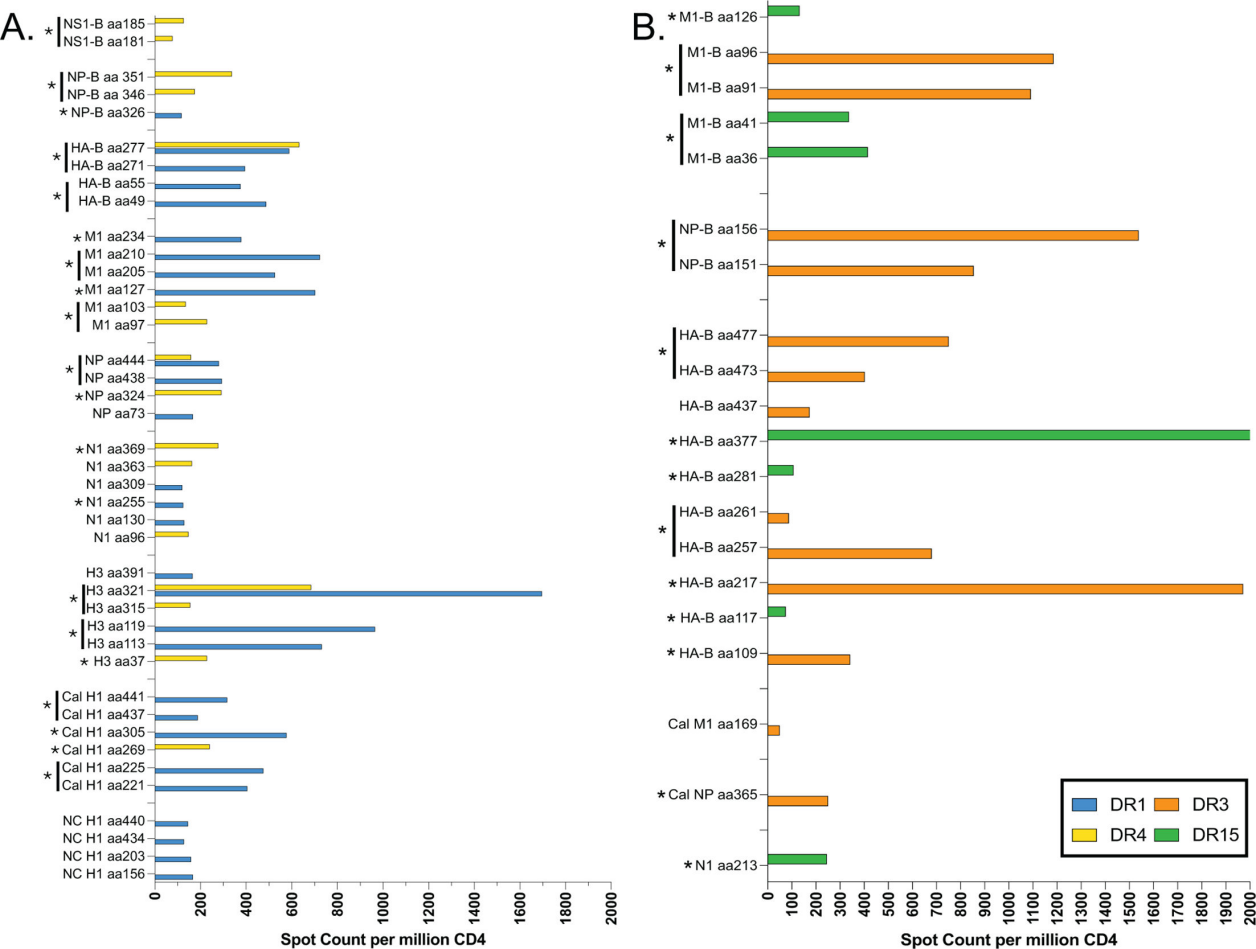
Summary of individual influenza H1N1, H3N2, and influenza B peptide-epitopes identified in HLA-DR transgenic mice. Shown are the average frequencies of cytokine-producing cells per million CD4 cells in HLA-DRB1*01:01 (“DR1,” blue) and HLA-DRB1*04:01 (“DR4,” yellow) transgenic mice in panel **A**, and HLA-DRB1*03:01 (“DR3,” orange) and HLA-DRB1*15:01 (“DR15,” green) transgenic mice in panel **B**. Epitopes that were tested in healthy adult human PBMC samples, shown in Fig. 10, are indicated with an asterisk. Adjacent overlapping peptides were tested as combined peptides and are indicated by a line next to the asterisk. Peptide epitope names have been indicated here with the protein and first amino acid number. The corresponding peptide number is shown in Tables 1 to 4.

Epitope discovery was initiated with either infection or vaccination of mice, depending on the virus and availability of recombinant proteins for vaccination, where CD4 T cells were tested *ex vivo* directly at the peak of the primary response. Shown in Fig. 1 is a schematic diagram, showing the generalized experimental path to identify single peptides from an average of approximately 400–600 potential peptides from influenza and SARS, respectively. An example of the first stages of this is shown in Fig. 2A for infection of HLA-DRB1*03:01 and HLA-DRB1*15:01 mice with influenza B virus (IBV). The focus of the CD4 T-cell response was initially assessed by quantifying cytokine producing cells to overlapping peptide pools encompassing the entire translated sequence of the individual candidate proteins (HA, NA, M1, NP, and NS1), using cytokine ELISpots. Using this approach, we found that for HLA-DRB1*03:01 (Fig. 2B), the response focused equally on HA, NP, and M1, with minimal responses to NA and NS1. These negative responses enable the elimination of proteins from further analyses. In contrast, the response in HLA-DRB1*15:01 mice to IBV was dominated by HA, representing almost 75% of the total response, with negligible responses to NP and NS1 and only a minor response to M1 (Fig. 2B). Thus, NA, NP, and NS1 were eliminated from consideration, and single peptide epitopes were defined for the dominant influenza virus proteins. Figure 2C illustrates the results from a peptide pooling matrix strategy for HA-B for HLA-DRB1*03:01 and HLA-DRB1*15:01 (Fig. 2D), where peptides are pooled so that no adjacent peptides are in the same pool of peptides (33). This strategy enables elimination of many peptides from further consideration (in gray) and identification of peptides in the most stimulatory pools (in yellow). The peptide candidates were tested in subsequent experiments shown in Fig. 3, allowing identification of several dominant peptides (e.g., HA p55) with the shorthand peptide nomenclature used to coincide with the matrix data (see Tables 3 and 4) and several minor peptides restricted to HLA-DR3 (Fig. 3) and one major HA peptide restricted to HLA-DR15 (HA p95) (Fig. 3B), with one or more minor epitopes. For smaller proteins, such as M1, single peptides were individually tested and confirmed in at least two experiments. Figure 3C and D shows the results for M1 for HLA-DR3 and HLA-DR15, where each of the peptides in the peptide array was tested. Epitope discovery was continued using these strategies for IAV. The summary of the epitope discovery for influenza virus responses is shown in Fig. 4 and individual peptide epitopes with sequences are listed in Tables 1 to 4, for each of the four HLA-DR mouse strains, that indicate the peptide number indicated in each array (e.g., “pX”), the starting and ending amino acid, the sequence of each peptide, and the method by which epitopes were mapped. Some peptides from our previous influenza studies in HLA-DR1 or HLA-DR4 mice (6, 34–39) were included for further examination for the ability to stimulate human CD4 T cells, and these are shown in Table 5.

**TABLE 1:** HLA-DRB1*01:01 (HLA-DR1) peptide epitope sequences with amino acid numbers, peptide names, and numbers used in the figures, and the method of priming used to map the peptide epitopes in influenza B and SARS-CoV-2.

HLA class II restriction	Virus	Method of priming	Protein	Peptide number	Peptide name	Amino acid sequence
HLA-DRB1*01:01	Influenza B	B/Brisbane and B/Florida infections	HA-B	HA-B p9	HA-B aa49	49 TTPTKSYFANLKGTRTR 65
				HA-B p10	HA-B aa55	55 YFANLKGTRTRGKLCPD 71
				HA-B p46	HA-B aa271	271 KTGTIVYQRGVLLPQKV 287
				HA-B p47	HA-B aa277	277 YQRGVLLPQKVWCASGR 293
			NP-B	NP-B p66	NP-B aa326	326 PSVASKVVLPISIYAKI 342
	SARS-CoV-2	Protein vaccination	Nucleocapsid	NCP p35	NCP aa171	171 FYAEGSRGGSQASSRSS 187
				NCP p36	NCP aa176	176 SRGGSQASSRSSSRSRN 192
			Helicase	NSP13 p39	NSP13 aa191	191 SKVQIGEYTFEKGDYGD 207
				NSP13 p40	NSP13 aa196	196 GEYTFEKGDYGDAVVYR 212
				NSP13 p45	NSP13 aa221	221 VGDYFVLTSHTVMPLSA 237
				NSP13 p56	NSP13 aa276	276 KYSTLQGPPGTGKSHFA 292
			NSP1	NSP1 p14	NSP1 aa66	66 QPYVFIKRSDARTAPHG 72
			NSP5	NSP5 p21	NSP5 aa88	88 LKVDTANPKTPKYKFVR 104
				NSP5 p47	NSP5 aa218	218 FLNRFTTTLNDFNLVAM 234
				NSP5 p51	NSP5 aa238	238 YEPLTQDHVDILGPLSA 254
			NSP7	NSP7 p5	NSP7 aa10	10 SVVLLSVLQQLRVESSS 26
				NSP7 p6	NSP7 aa15	15 SVLQQLRVESSSKLWAQ 31
				NSP7 p11	NSP7 aa40	40 LLAKDTTEAFEKMVSLL 56
				NSP7 p13	NSP7 aa50	50 EKMVSLLSVLLSMQGAV 66
				NSP7 p14	NSP7 aa55	55 LLSVLLSMQGAVDINKL 71
			NSP8	NSP8 p31	NSP8 aa138	138 YKNTCDGTTFTYASALW 154
				NSP8 p32	NSP8 aa143	143 DGTTFTYASALWEIQQV 159
				NSP8 p38	NSP8 aa173	173 SMDNSPNLAWPLIVTAL 189
			Spike	Spk p64	Spk aa316	316 SNFRVQPTESIVRFPNI 332
				Spk p97	Spk aa481	481 NGVEGFNCYFPLQSYGF 497
				Spk p98	Spk aa486	486 FNCYFPLQSYGFQPTNG 502
				Spk p103	Spk aa511	511 VVLSFELLHAPATVCGP 527
				Spk p109	Spk aa541	541 FNFNGLTGTGVLTESNK 557
				Spk p118	Spk aa586	586 DITPCSFGGVSVITPGT 602

**TABLE 2:** HLA-DRB1*04:01 (HLA-DR4) peptide epitope sequences with amino acid numbers, peptide names, and numbers used in the figures, and the method of priming used to map the peptide epitopes in influenza B and SARS-CoV-2

HLA class II restriction	Virus	Method of priming	Protein	Peptide number	Peptide name	Amino acid sequence
HLA-DRB1*04:01	Influenza B	B/Brisbane and B/Florida infections	HA-B	HA-B p47	HA-B aa277	277 YQRGVLLPQKVWCASGR 293
			NP-B	NP-B p70	NP-B aa346	346 GFNVEEYSMVGYEAMAL 362
				NP-B p71	NP-B aa351	351 EYSMVGYEAMALLYNMAT 367
			NS1-B	NS1-B p46	NS1-B aa181	181 NVLSLRVLVNGTFLKH 196
				NS1-B p47	NS1-B aa185	185 LRVLVNGTFLKHPNGY 200
	SARS-CoV-2	Protein vaccination	Helicase	NSP13 p26	NSP13 aa126	126 CTERLKLFAAETLKATE 142
				NSP13 p27	NSP13 aa131	131 KLFAAETLKATEETFKL 147
				NSP13 p28	NSP13 aa136	136 ETLKATEETFKLSYGIA 152
				NSP13 p79	NSP13 aa391	391 LRAKHCVYIGDPAQLPA 407
				NSP13 p109	NSP13 aa541	541 YDYVIFTQTTETAHSCN 557
			NSP5	NSP5 p30	NSP5 aa133	133 FTIKGSFLNGSCGSVGF 149
				NSP5 p31	NSP5 aa138	138 SFLNGSCGSVGFNIDYD 154
			NSP7	NSP7 p11	NSP7 aa40	40 LLAKDTTEAFEKMVSLL 56
			NSP8	NSP8 p31	NSP8 aa138	138 YKNTCDGTTFTYASALW 154
				NSP8 p32	NSP8 aa143	143 DGTTFTYASALWEIQQV 159
				NSP8 p37	NSP8 aa168	168 QLSEISMDNSPNLAWPL 184
			Spike	Spk p8	Spk aa36	36 VYYPDKVFRSSVLHSTQ 52
				Spk p57	Spk aa281	281 ENGTITDAVDCALDPLS 297
				Spk p92	Spk aa456	456 FRKSNLKPFERDISTEI 472
				Spk p93	Spk aa461	461 LKPFERDISTEIYQAGS 477
				Spk p161	Spk aa801	801 NFSQILPDPSKPSKRSF 817

**TABLE 3:** HLA-DRB1*03:01 (HLA-DR3) peptide epitope sequences with amino acid numbers, peptide names, and numbers used in the figures, and the method of priming used to map the peptide epitopes in influenza B and SARS-CoV-2.

HLA class II restriction	Virus	Method of priming	Protein	Peptide number	Peptide name	Amino acid sequence
HLA-DRB1*03:01	Influenza B	B/Brisbane infection	HA-B Brisbane	HA-B p28	HA-B aa109	109 CFPIMHDRTKIRQLP 123
				HA-B p55	HA-B aa217	217 AKLYGDSKPQKFTSS 231
				HA-B p65	HA-B aa257	257 QSGRIVVDYMVQKSG 271
				HA-B p66	HA-B aa261	261 IVVDYMVQKSGKTGT 275
				HA-B p110	HA-B aa437	437 NEILELDEKVDDLRA 451
				HA-B p119	HA-B aa473	473 EDEHLLALERKLKKM 487
				HA-B p120	HA-B aa477	477 LLALERKLKKMLGPS 491
			NP-B	NP-B p31	NP-B aa151	151 GGTFYKMVRDDKTIYFS 167
				NP-B p32	NP-B aa156	156 KMVRDDKTIYFSPIRIT 172
			M1-B	M1-Bp19	M1-B aa91	91 TKKKGLILAERKMRRCV 107
				M1-Bp20	M1-B aa96	96 LILAERKMRRCVSFHEA 112
	SARS-CoV-2	Protein vaccination	Nucleocapsid	NCP p32	NCPaa156	156 AIVLQLPQGTTLPKGFY 172
			Helicase	NSP13 p31	NSP13 aa151	151 IATVREVLSDRELHLSW 167
				NSP13 p32	NSP13 aa156	156 EVLSDRELHLSWEVGKP 172
				NSP13 p90	NSP13 aa446	446 AEIVDTVSALVYDNKLK 462
				NSP13 p91	NSP13 aa451	451 TVSALVYDNKLKAHKDK 467
				NSP13 p92	NSP13 aa456	456 VYDNKLKAHKDKSAQCF 472
			Spike	Spk p60	Spk aa296	296 LSETKCTLKSFTVEKGI 312
				Spk p72	Spk aa356	356 KRISNCVADYSVLYNSA 372
				Spk p80	Spk aa396	396 YADSFVIRGDEVRQIAP 412
				Spk p81	Spk aa401	401 VIRGDEVRQIAPGQTGK 417
				Spk p161	Spk aa801	801 NFSQILPDPSKPSKRSF 817
				Spk p217	Spk aa1081	1081 ICHDGKAHFPREGVFVS 1097

**TABLE 4:** HLA-DRB1*15:01 (HLA-DR15) peptide epitope sequences with amino acid numbers, peptide names, and numbers used in the figures, and the method of priming used to map the peptide epitopes in influenza B and SARS-CoV-2.

HLA class II restriction	Virus	Method of priming	Protein	Peptide number	Peptide name	Amino acid sequence
HLA-DRB1*15:01	Influenza B	B/Brisbane infection	HA-B Brisbane	HA-B p30	HA-B aa117	117 TKIRQLPNLLRGYEH 131
				HA-Bp71	HA-B aa281	281 GILLPQKVWCASGRS 295
				HA-Bp95	HA-B aa377	377 EGMIAGWHGYTSHGA 391
			M1-B	M1-Bp8	M1-B aa36	36 EFDLDSALEWIKNKRCL 52
				M1-Bp9	M1-B aa41	41 SALEWIKNKRCLTDIQK 57
				M1-Bp26	M1-B aa126	126 YCLMVMYLNPGNYSMQV 142
	SARS-CoV-2	Protein vaccination	Nucleocapsid	NCP p57	NCPaa281	281 QTQGNFGDQELIRQGTD 297
			Helicase	NSP13 p18	NSP13 aa86	86 NGQVFGLYKNTCVGSDN 102
				NSP13 p59	NSP13 aa291	291 FAIGLALYYPSARIVYT 307
				NSP13 p75	NSP13 aa371	371 VVFDEISMATNYDLSVV 387
				NSP13 p93	NSP13 aa461	461 LKAHKDKSAQCFKMFYK 477
				NSP13 p102	NSP13 aa506	506 WRKAVFISPYNSQNAVA 522
				NSP13 p103	NSP13 aa511	511 FISPYNSQNAVASKILG 527
			Spike	Spk p87	Spk aa431	431 GCVIAWNSNNLDSKVGG 447
				Spk p150	Spk aa746	746 STECSNLLLQYGSFCTQ 762
				Spk p151	Spk aa751	751 NLLLQYGSFCTQLNRAL 767

**TABLE 5:** Influenza peptide epitope sequences with amino acid numbers and peptide names that correspond to the peptides assessed for CD4 T-cell reactivity in healthy adults shown in Fig. 10A

HLA restriction	Peptide name^*a*^	Sequence
HLA-DRB1*01:01	Cal H1 aa221 (37)	221 SRYSKKFKPEIAIRP 235 225 KKFKPEIAIRPKVRD 239
	Cal H1 aa305 (37)	305 TSLPFQNIHPITIGK 319
	Cal H1 aa437 (37)	437 TYNAELLVLLENERT 451 441 ELLVLLENERTLDYH 455
	NA aa255 (34)	255 IFKIEKGKVTKSIELNA 271
	H3 aa113 (37)	113 CYPYDVPDYASLRSLVA 129 119 PDYASLRSLVASSGTLE 135
	H3 aa321 (37)	321 CPRYVKQNTLKLATGMR 337
	NP aa438 (34)	438 SDMRAEIIKMMESARPE 454 444 IIKMMESARPEEVSFQG 460
	M1 aa127 (34)	127 CMGLIYNRMGAVTTESA 143
	M1 aa205 (34)	205 VASQARQMVQAMRAIGT 221 210 RQMVQAMRAIGTHPSSS 226
	M1 aa234 (34)	234 LENLQAYQKRMGVQMQR 250
	HA-B aa49	49 TTPTKSYFANLKGTRTR 65 55 YFANLKGTRTRGKLCPD 71
	HA-B aa271	271 KTGTIVYQRGVLLPQKV 287 277 YQRGVLLPQKVWCASGR 293
	NP-B aa326	326 PSVASKVVLPISIYAKI 342
HLA-DRB1*04:01	Cal H1 aa269 (37)	269 RYAFAMERNAGSGII 283
	NC NA aa369 (36)	369 KGFEMIWDPNGWTDTDS 385
	Cal NA aa369	369 KGFEMIWDPNGWTDT 383
	H3 aa37 (37)	37 PNGTIVKTITNDQIEVT 53
	H3 aa321 (37)	315 RITYGACPRYVKQNTLK 331 321 CPRYVKQNTLKLATGMR 337
	NP aa324 (36)	324 HKSQLVWMACNSAAFED 340
	NP aa444 (36)	444 IIKMMESARPEEVSFQG 460
	M1 aa97 (36)	97 VKLYRKLKREITFHGAK 113 103 LKREITFHGAKEIALSY 119
	HA-B aa277	277 YQRGVLLPQKVWCASGR 293
	NP-B aa346	346 GFNVEEYSMVGYEAMAL 362 351 EYSMVGYEAMALLYNMAT 367
	NS1-B aa181	181 NVLSLRVLVNGTFLKH 196 185 LRVLVNGTFLKHPNGY 200
HLA-DRB1*03:01	NP aa365	365 IASNENVETMDSNTL 379
	HA-B aa109	109 CFPIMHDRTKIRQLP 123
	HA-B aa217	217 AKLYGDSKPQKFTSS 231
	HA-B aa257	257 QSGRIVVDYMVQKSG 271 261 IVVDYMVQKSGKTGT 275
	HA-B aa473	473 EDEHLLALERKLKKM 487 477 LLALERKLKKMLGPS 491
	NP-B aa151	151 GGTFYKMVRDDKTIYFS 167 156 KMVRDDKTIYFSPIRIT 172
	M1-B aa91	91 TKKKGLILAERKMRRCV 107 96 LILAERKMRRCVSFHEA 112
HLA-DRB1*15:01	NA aa213	213 TDTIKSWRNNILRTQ 227
	HA-B aa117	117 TKIRQLPNLLRGYEH 131
	HA-B aa281	281 GILLPQKVWCASGRS 295
	HA-B aa377	377 EGMIAGWHGYTSHGA 391
	M1-B aa36	36 EFDLDSALEWIKNKRCL 52 41 SALEWIKNKRCLTDIQK 57
	M1-B aa126	126 YCLMVMYLNPGNYSMQV 142

^*a*^ References for peptide epitopes previously identified by our group are indicated next to the peptide name.

**Fig 5:**
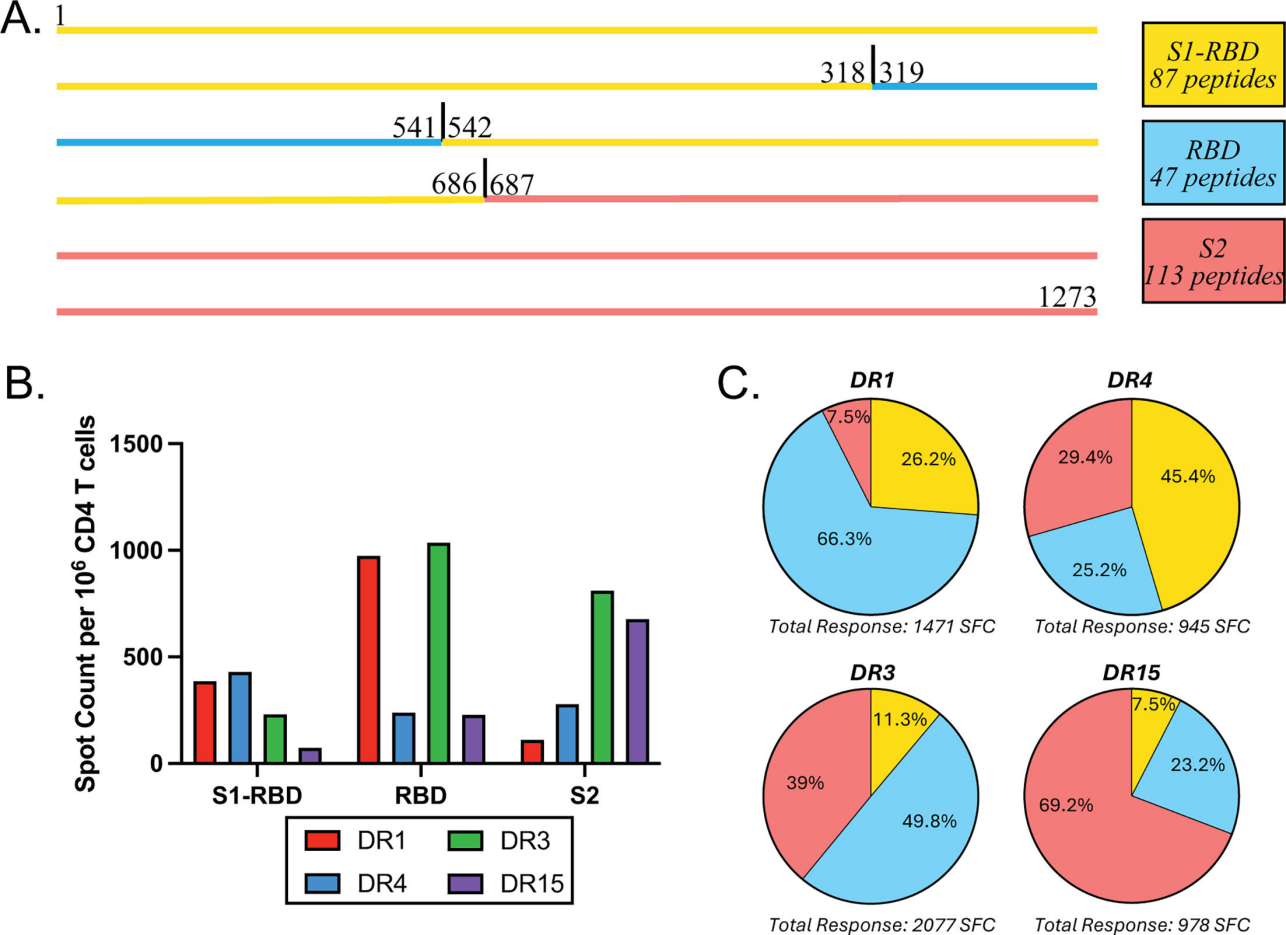
HLA-DR transgenic mice have distinct immunodominance patterns to SARS-CoV-2 spike protein. The responses to recombinant spike protein were evaluated in ELISpot T assays following vaccination with SARS-CoV-2 spike, using overlapping peptide pools representing each segment (shown in panel **A**), indicated in the colored boxes to the right. (**B**) CD4 T-cell responses in HLA-DR1 (red), DR4 (blue), DR3 (green), and DR15 (purple) mice that had been vaccinated with recombinant SARS-CoV-2 spike protein as described in Materials and Methods. Responses are shown as the frequency of IL-2-producing cells per million CD4 T cells for each of the overlapping peptide libraries indicated in panel **A**. Panel **C** illustrates the percent of the response to each segment of spike (S1-RBD [yellow], RBD [blue], and S2 [salmon]) and the total response to SARS-CoV-2 spike protein indicated below each pie.

**Fig 6:**
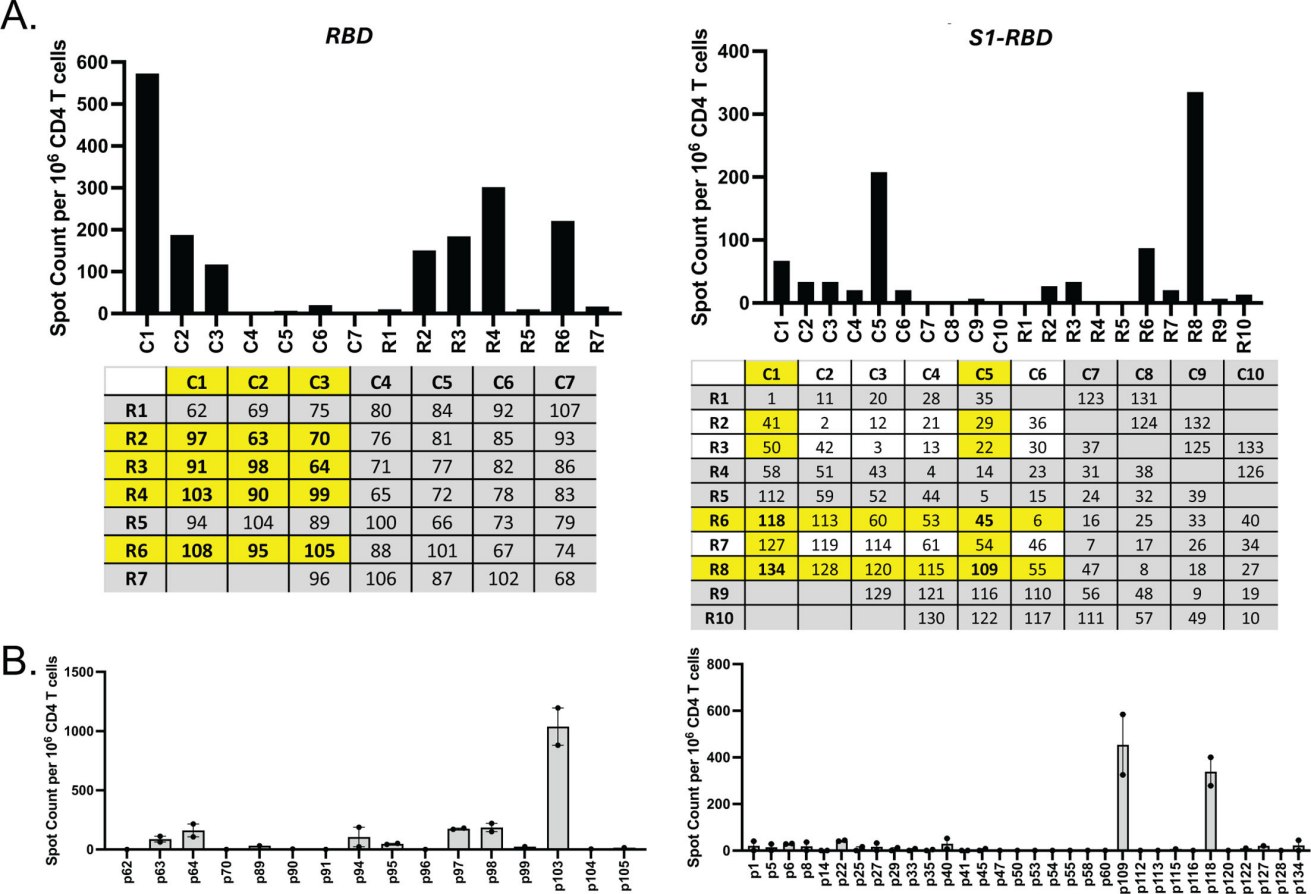
Mapping single peptide epitopes in SARS-CoV-2 spike protein using matrices. Example of matrices for SARS-CoV-2 spike in HLA-DR1 mice using the immunodominant subdomains identified as shown in Fig. 5 (**A**; RBD, left, and S1-RBD, right). Candidate single peptides from the matrix, highlighted in yellow, were tested in the subsequent assay shown in panel **B**.

**Fig 7:**
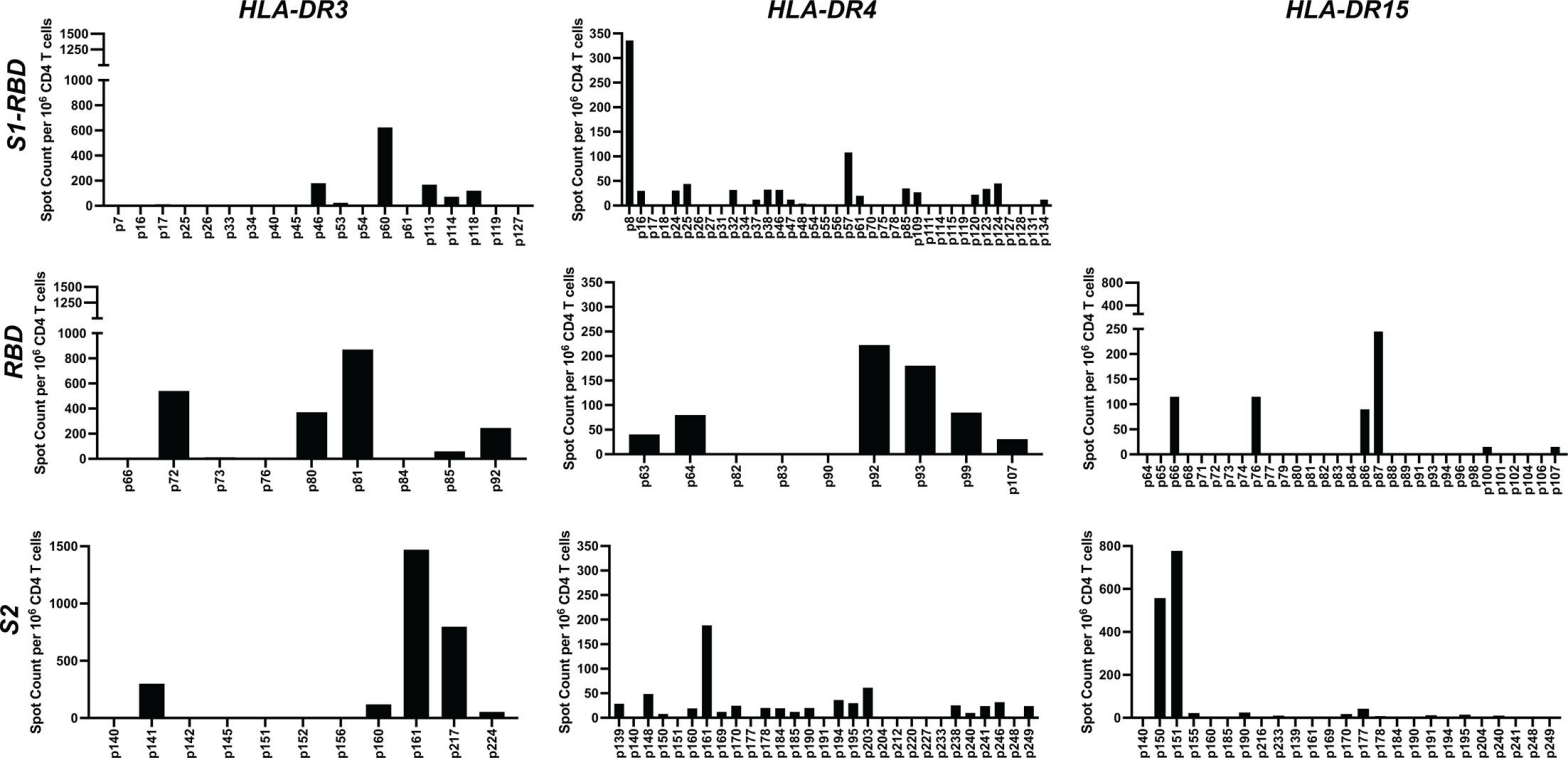
Single peptide analysis of the spike protein for HLA-DR3, HLA-DR4, and HLA-DR15. An example of single peptide analyses is shown following vaccination of the indicated HLA-DR transgenic with SARS-CoV-2 spike protein. Candidate peptides were identified based on the results of peptide pooling matrices (not shown, representative example shown in Fig. 6). There was negligible reactivity to epitopes contained in the S1-RBD segment in HLA-DR15 transgenic.

**Fig 8:**
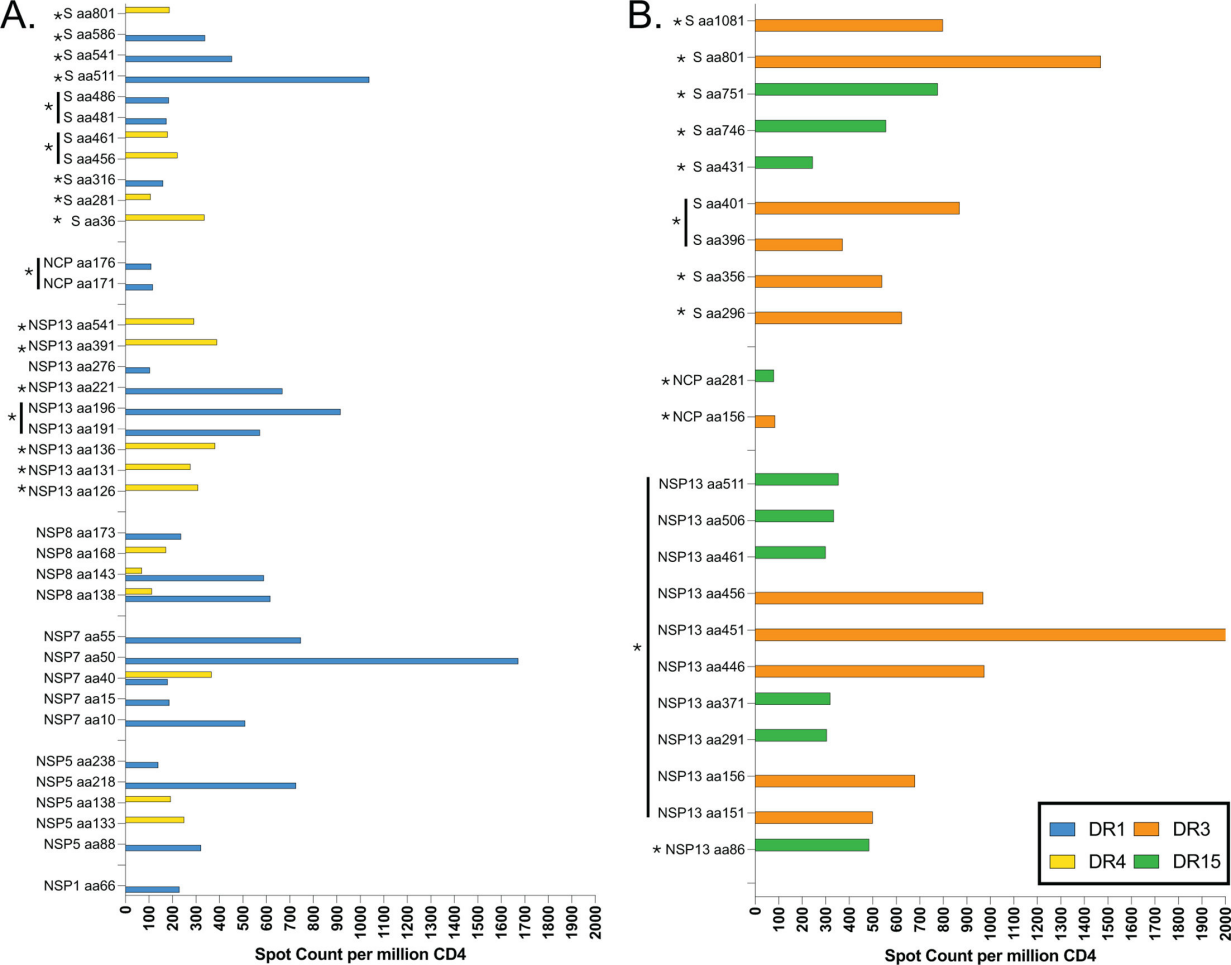
Summary of SARS-CoV-2 CD4 T-cell peptide epitopes identified in HLA-DR transgenic mice. Shown is the average frequency of cytokine-producing cells per million CD4 cells in HLA-DRB1*01:01 (“DR1,” blue [**A**]) and HLA-DRB1*04:01 (“DR4” [**A**]), HLA-DRB1*03:01 (“DR3” [**B**]), and HLA-DRB1*15:01 (“DR15,” green [**B**]) transgenic mice vaccinated with recombinant SARS-CoV-2 proteins. Epitopes that were tested in healthy adult human PBMC samples, shown in Fig. 10, are indicated with an asterisk. Adjacent overlapping peptides were tested as a combined peptide and are indicated by a line next to the asterisk. In some cases, helicase peptides were tested as a selected pool in assays assessing reactivity in human PBMC. Peptide epitope names have been indicated here with the protein and amino acid number. The corresponding peptide number is shown in Tables 1 to 4.

To extend epitope discovery to SARS-CoV-2, recombinant protein vaccination was used, followed as before by direct *ex vivo* ELISpot analyses. For Spike, a large protein consisting of over 250 overlapping peptides, the peptide libraries were separated into three domains for screening. The segments and boundaries across Spike are shown in Fig. 5A. The receptor-binding domain (“RBD”) consisted of 47 overlapping peptides, the S1 segment minus the RBD (“S1-RBD”) consisted of 87 peptides, and “S2,” which is the entire conserved membrane proximal domain, consisted of 119 overlapping peptides. Peptides at the boundaries of each segment were included in both pools. Figure 5B and C shows the results of testing reactivity to each of the three segments, after the HLA-DR transgenic mice were vaccinated with recombinant full-length SARS-CoV-2 spike protein, formulated with an adjuvant. The dominance observed was unique to each strain, indicating strong HLA-DR-linked epitope selection. DR1-restricted responses were dominated by RBD and S1-RBD-derived peptides, whereas DR3 responses were dominated by the smaller RBD and S2-derived peptide pools. These results were followed by the peptide pooling matrix analyses (Fig. 6A). The elimination/selection experiments were followed by single peptide analyses, shown in Fig. 6B. This iterative process enabled selection from 253 total spike-derived peptides to 6 peptides presented by HLA-DR1, 6 from HLA-DR3, 5 from HLA-DR4, and 3 from HLA-DR15**,** shown in Fig. 7, when using a frequency of 100 cytokine-producing cells per million as an “immunodominant positive” peptide. These studies were next extended to other SARS-CoV-2-derived peptides from nucleocapsid (N), helicase, and a subset of non-structural proteins (NSPs). The choices across SARS-CoV-2 proteins were in part based on protein availability and in part based on homology with seasonal human coronaviruses (HCoV), where amino acid sequence alignments suggested the potential for recall from T-cell memory established by these HCoV, prominent for helicase and N, which we anticipated could enrich reactivity in human CD4 T cells. Figure 8A and B show the results of epitope discovery in the 4 strains of HLA-DR transgenic mice for SARS-CoV-2-derived epitopes. Tables 1 to 4 indicate all the identified individual epitopes with peptide numbers, amino acid ranges, and sequences. It should be noted that different strains of mice exhibit characteristic relative immunogenicity in CD4 T-cell responses after infection or vaccination. These differences may relate to the site of integration of the transgene or selection of the CD4 T-cell repertoire in the host. We have no evidence that the HLA-DR transgenic mice differ in the fraction of cells that are positive for HLA-DR or the cell surface density of HLA-DR molecules.

**Fig 9:**
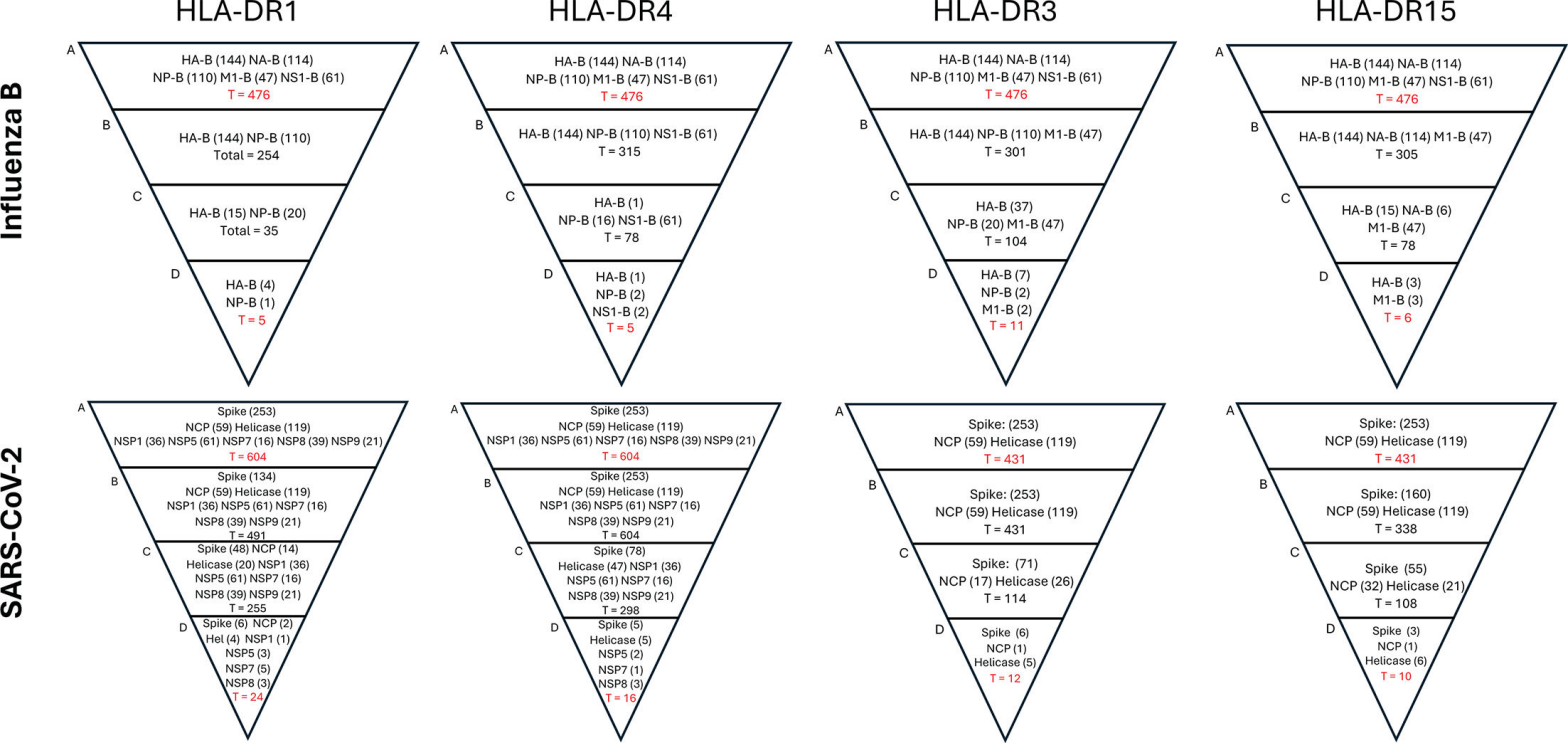
A funnel approach to CD4 T-cell epitope identification. Shown are funnel diagrams of the iterative process of epitope discovery for influenza B (top) or SARS-CoV-2 epitopes (bottom). In the top slice of each funnel (“A”), the total number of peptides screened for each individual HLA-DR allele or virus is indicated, with the proteins listed and the number of total peptides contained in each protein shown in parentheses. The total number of peptides is indicated in red (“T” = total). In slice “B,” narrowing of the potential candidates using overlapping pools of peptides is shown. If no proteins were eliminated by pools because pools were all positive, this value is the same as the value in slice “A.” In slice “C,” the potential single epitopes tested from either matrix or single peptides from small proteins such as NS1 or M1 are indicated. Finally, in slice “D,” the number of positive peptides from the single peptide analysis is indicated, and the total number of identified peptides is indicated in red (“T”). The individual peptides have been shown in the horizontal bar graphs in Fig. 4 and 8 and listed in Tables 1 to 4. These are the dominant CD4 peptide epitope candidates for applications in humans or further animal models.

In Fig. 9, we illustrate the process and benefit of the systematic and unbiased approach that we have described as a “funnel approach,” which is an iterative process involving sequential elimination and selection of the many candidate epitopes based on direct *ex vivo* assays, followed by determination of the single HLA-DR-restricted immunogenic peptides. This approach, which was schematized in Fig. 1 illustrating each “slice” of the funnel, can be used with any pathogen of interest from which protein sequences are known, where the investigator seeks to identify single HLA-restricted epitopes. The top slice of the funnel (A) shows the potential number of peptide epitopes from all the proteins that were tested in each HLA-DR transgenic mouse strain, based on the number of overlapping peptides in each of the influenza B (Fig. 9A) or SARS-CoV-2 (Fig. 9B) proteins tested. Some mouse strains were not tested by IAV infection because of high pathogenicity. The sequential slices of the funnel indicate the number of peptides that remain at each stage after testing the total pooled peptides, eliminating those in pools that elicited fewer than 50 spots (B). For large proteins (HA, NP, and NA) that were positive based on total pools, peptide pooling matrices enabled further elimination of peptides based on non-stimulatory peptide pools in the matrices (C). These sequential processes left single candidate peptides from HA, NP, and NA, as well as the complete set of single overlapping peptides from small proteins (NS1 and M1) that were tested individually, allowing further elimination of non-immunogenic peptides (D). This process yielded immunogenic, HLA-DR-restricted peptide candidates that could then be used to test in HLA-typed human CD4 T cells. Depending on the HLA-DR allele, this process enriched candidates from 400 to 600 to approximately 5–24, depending on the pathogen and the HLA-DR allele. Combining all four alleles for HLA-DR, a total of 27 epitopes were discovered from IBV using this approach, and 62 peptides from SARS-CoV-2.

### Testing of reactivity in HLA-typed human subjects

After identification of peptide epitopes using the HLA-DR transgenic mice, we evaluated whether the single peptides were sufficiently immunogenic in humans to be detected directly *ex vivo*, without any expansion in culture, a common strategy used by other groups (30, 40, 41) due to the low frequencies of single epitope-specific T cells from human PBMC. We surveyed the single HLA-DR-restricted epitopes identified from circulating CD4 T cells from healthy adults with no reported history of infection or vaccination, thus likely reflecting the memory compartment. We focused on peptides that we expected to be useful for human studies, such as those derived from surface receptors (influenza HA and SARS-CoV-2 spike), that are the target of vaccinations, or epitopes from internal virion proteins that are genetically conserved.

**Fig 10:**
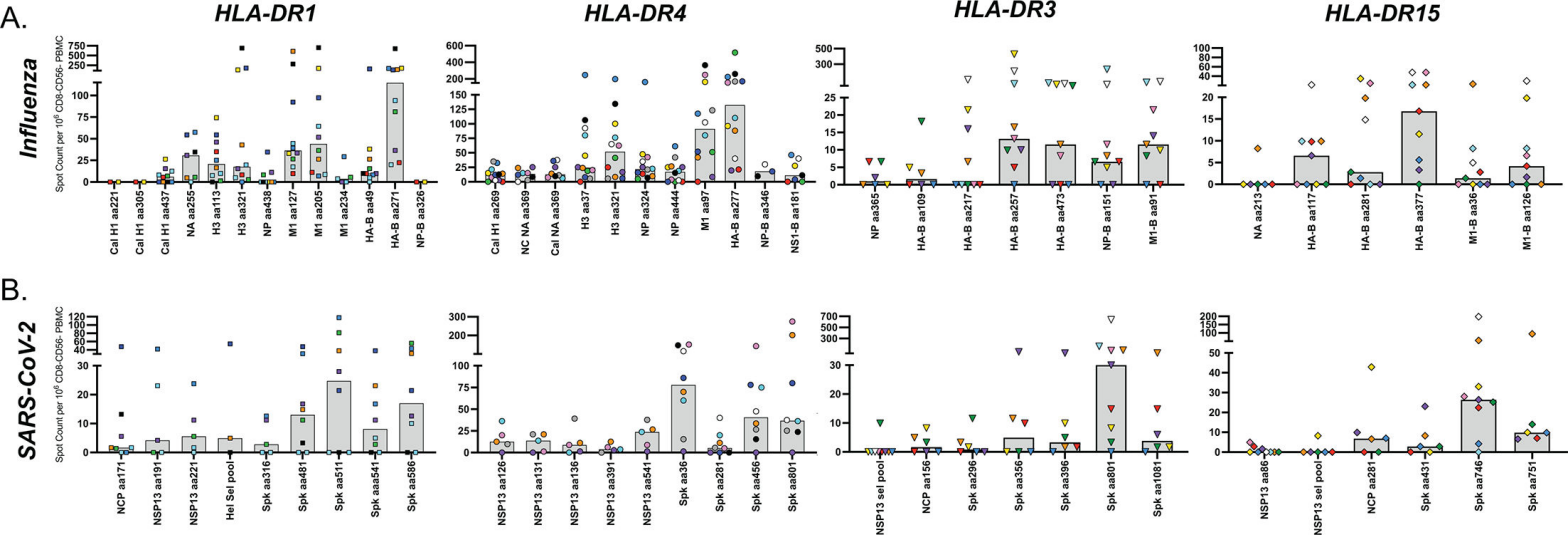
Human CD4 T responses to influenza H1-, H3-, B-, and SARS-CoV-2-derived epitopes. PBMCs from healthy adults that were collected between 2018 and 2023 and typed for HLA-DRB1*01:01 (far left), HLA-DRB1*04:01 (left middle), HLA-DRB1*03:01 (right middle), or HLA-DRB1*15:01 (far right) were enriched for CD4 T cells and APC by depletion of CD8+ and CD56+ cells. CD4 T-cell reactivity to each of the individual influenza peptides (**A**) and SARS-CoV-2 peptides (**B**) that were previously identified by epitope mapping in HLA-DR transgenic were assessed in IFN-γ ELISpot assays. Individual subjects are represented by unique symbols, and the median response is shown as a gray bar. All data are represented as spots per million CD4 T cell-enriched PBMC, with background subtracted. Demographics and unique symbols for each subject are shown in Table S3, peptide sequences are shown in Tables 5 and 6, and the full HLA-typing for each subject is shown in Table S4.

To assess single epitope-specific cells in humans, CD4 T cells were enriched from cryopreserved PBMC by depletion of CD8 T cells and NK cells (which can also produce IFN-γ), leaving APC and CD4 T cells primarily with <1% CD8 T cells. These CD4 T-cell-enriched populations were tested with the single dominant peptides identified using HLA-DR transgenic mice. Tables 5 and 6 show the sequences of the peptides tested in the human CD4 T cells, from these studies or previously published work from our laboratory in DR1 and DR4 using analogous approaches as described here for epitope discovery using HLA-DR transgenic mice (6, 34–37). For overlapping peptides that might represent a single epitope, the two peptides were combined for stimulation of the human CD4 T cells, as indicated in Tables 5 and 6. HLA-typed donors, consisiting of 6–12 donors, were tested for each allele and epitope. Figure 10 (top) shows the results with HLA-DR typed donors tested with influenza-derived peptide epitopes identified from N1, M1, NP, and HA proteins. Our data revealed that the abundance of CD4 T-cell epitope-specific cells varied among subjects, likely reflecting both the infection and vaccination history, and inherent subject-dependent magnitude and phenotype shown to be predicted even before influenza vaccination (42). For example, some HLA-DR1 subjects exhibited relatively robust responses to many epitopes, including those derived from influenza HA (HA-B aa271 p46/47) and M1 (M1 aa127 and aa205), and other subjects were modest in the frequency of those single peptide-specific CD4 T cells detected. When HLA-DR4, HLA-DR3, and HLA-DR15 subjects were probed in a similar way, reactivity to single peptides derived from H1, H3, HA-B, M1, NP, and NS1 were evaluated, from which many were also confirmed.

**TABLE 6:** SARS-CoV-2 peptide epitope sequences with amino acid numbers and peptide names that correspond to the peptides assessed for CD4 T-cell reactivity in healthy adults shown in Fig. 10B

HLA restriction	Peptide name	Sequence
HLA-DRB1*01:01	NCP aa171	171 FYAEGSRGGSQASSRSS 187 176 SRGGSQASSRSSSRSRN 192
	NSP13 aa191	191 SKVQIGEYTFEKGDYGD 207 196 GEYTFEKGDYGDAVVYR 212
	NSP13 aa221	221 VGDYFVLTSHTVMPLSA 237
	Hel Sel Pool	All NSP13 peptides listed in Table 1
	Spk aa316	316 SNFRVQPTESIVRFPNI 332
	Spk aa481	481 NGVEGFNCYFPLQSYGF 497 486 FNCYFPLQSYGFQPTNG 502
	Spk aa511	511 VVLSFELLHAPATVCGP 527
	Spk aa541	541 FNFNGLTGTGVLTESNK 557
	Spk aa586	586 DITPCSFGGVSVITPGT 602
HLA-DRB1*04:01	NSP13 aa126	126 CTERLKLFAAETLKATE 142
	NSP13 aa131	131 KLFAAETLKATEETFKL 147
	NSP13 aa136	136 ETLKATEETFKLSYGIA 152
	NSP13 aa391	391 LRAKHCVYIGDPAQLPA 407
	NSP13 aa541	541 YDYVIFTQTTETAHSCN 557
	Spk aa36	36 VYYPDKVFRSSVLHSTQ 52
	Spk aa281	281 ENGTITDAVDCALDPLS 297
	Spk aa456	456 FRKSNLKPFERDISTEI 472 461 LKPFERDISTEIYQAGS 477
	Spk aa801	801 NFSQILPDPSKPSKRSF 817
HLA-DRB1*03:01	Hel Sel Pool	All NSP13 peptides listed in Table 3
	NCPaa156	156 AIVLQLPQGTTLPKGFY 172
	Spk aa296	296 LSETKCTLKSFTVEKGI 312
	Spk aa356	356 KRISNCVADYSVLYNSA 372
	Spk aa396	396 YADSFVIRGDEVRQIAP 412 401 VIRGDEVRQIAPGQTGK 417
	Spk aa801	801 NFSQILPDPSKPSKRSF 817
	Spk aa1081	1081 ICHDGKAHFPREGVFVS 1097
HLA-DRB1*15:01	NSP13 aa86	86 NGQVFGLYKNTCVGSDN 102
	Hel Sel Pool	All NSP13 peptides listed in Table 4
	NCP aa281	281 QTQGNFGDQELIRQGTD 297
	Spk aa431	431 GCVIAWNSNNLDSKVGG 447
	Spk aa746	746 STECSNLLLQYGSFCTQ 762
	Spk aa751	751 NLLLQYGSFCTQLNRAL 767

The ability of the SARS-CoV-2-derived peptides identified in HLA-DR transgenic mice to stimulate IFN-γ production was then examined in human CD4 T cells (Fig. 10, bottom). Most donors exhibited detectable and sometimes robust reactivity to several peptides, particularly to spike-derived single peptides. For example, HLA-DR1 subjects have a robust response to Spike aa511 and HLA-DR4 subjects to Spike aa36. This is not surprising, as Spike specificities are elicited by infection and vaccination. Interestingly, CD4 T-cell reactivity was also detected to some non-spike epitopes, including Nucleocapsid (N) and Helicase, suggesting that these were induced by infection. Individuals displayed unique patterns, with some peptides eliciting robust responses while displaying little reactivity to others, indicating that the cytokine responses detected were epitope-specific. The HLA-DR-restricted specificities identified here provide candidates for further studies.

### Tetramer staining enriches CD4 T-cell memory populations from PBMC

**Fig 11:**
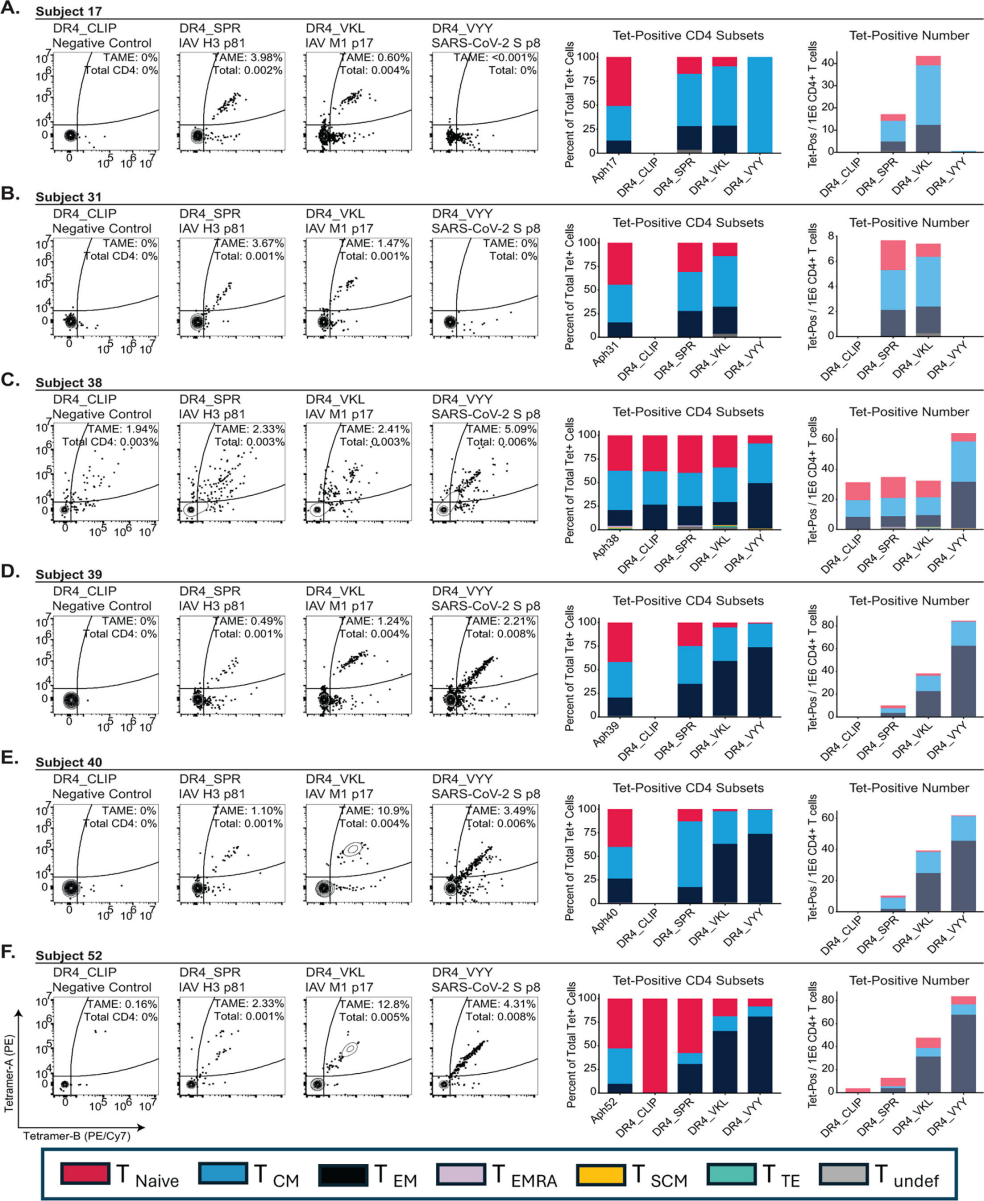
. Characterization of human CD4+ T subsets following tetramer-associated magnetic enrichment. (**A–F**) PBMCs from donors with HLA-DRB1*04:01 (full HLA typing shown in Table S5) were dual-stained with PE- and PE/Cy7-conjugated HLA-DR4 tetramers loaded with CLIP (negative control), IAV H3 p81 (SPRYVKQNTLKLATGMR), IAV/IBV M1 p17 (VKLYRKLKREITFHGAK), or SARS-CoV-2 S p8 (VYYPDKVFRSSVLHSTQ) and subjected to TAME. TAME-selected cells were surface stained with a CD4 immunophenotyping panel and analyzed by flow cytometry. Flow plots depict percentages of cells positive for tetramer labeled with both colors (Tet+) as a percentage of TAME-selected (top; inset) and total (bottom; inset) live CD3+ CD4+ lymphocytes. The composition of Tet+ CD4+ T-cell subsets is depicted in the bar graphs as either the percentage of total Tet+ cells for a given tetramer (middle) or Tet+ counts per 1 × 10^6^ CD4+ T cells (far right). The composition of the total CD4+ populations (non-TAME) for each subject is provided (left bar). Bar graph positions with no values indicate that no Tet+ cells were identified. CD4+ T-cell subsets included Tnaïve (naïve); Tcm (central memory), Tem (effector memory), T_emra_ (terminally Temra; effector memory CD45RA positive), Tscm (stem cell memory), Tte (terminally exhausted), and T undefined (not defined by other listed categories).

To further evaluate the funnel approach in identifying viral epitopes, we next determined whether selected peptide:HLA tetramers could enrich CD4 T-cell responses directly from human PBMC. HLA-DR4 molecules were assembled with the candidate IAV H3 p81, IAV M1 p17, and SARS-CoV-2 p8 peptides to evaluate human CD4 T cells specific for both influenza A and SARS-CoV-2. Negative control DR4 tetramers bound to the CLIP peptide were also generated. PBMCs from six healthy adult donors with confirmed HLA-DRB1*04:01 were then stained with tetramers and enriched using tetramer-associated magnetic enrichment (TAME). Tetramer staining with the DR4 CLIP tetramers indicated minimal background staining. By testing with the same tetramer conjugated with alternate fluorochromes, we were able to quantify the double-positive CD4 T cells with confidence. Using this strategy, circulating CD4 T cells positive for IAV H3 p81 and M1 p17 were detected in all donor samples (Fig. 11, left). SARS-CoV-2 S p8 tetramer-positive CD4 T cells were only detected in donors sampled after 2019 (donors 38, 39, 40, and 52), while no SARS-CoV-2 Sp8 tetramer-positive CD4 T cells were detected in pre-2019 donors 17 and 31 (Fig. 11, left). To determine the composition of the epitope-specific CD4 T cells, the tetramer-positive fractions and unenriched PBMC samples were surface stained with a CD4 memory phenotyping flow cytometry panel. Compared to the unenriched PBMCs, tetramer-positive CD4 T cells were enriched for central memory (TCM) and effector memory (TEM) populations while minimizing naive T-cell staining in both frequency of TAME-enriched CD4 T cells (Fig. 11, left bar plot) and number per million CD4 T cells (Fig. 11, right bar plot). Together, these data support the funnel approach as a valuable strategy for human virus epitope discovery that can enable the generation of tetramers capable of enriching and analyzing CD4 T-cell memory populations directly from donor PBMCs.

## DISCUSSION

In this study, we describe an iterative process for defining HLA-DR-restricted epitopes, using progressive cycles of elimination and selection of robust CD4 T-cell epitopes directly *ex vivo* in mice and then extend this to direct *ex vivo* analyses of human CD4 T cells, also analyzed without any expansion of cells *in vitro*. This process enabled the identification of a diverse set of CD4 T-cell epitopes from multiple proteins from the respiratory pathogens Influenza A, Influenza B, and SARS-CoV-2. Often, CD4 T-cell immunity to emerging pathogens such as SARS-CoV-2 or the novel pandemic H1N1 strain of influenza has been evaluated in both our own laboratory (43–46) and others’ (47–50) (see reviews in references 51, 52), using large peptide pools representing the translated sequence of a pathogen-derived protein. Although very useful to quantify the full response magnitude of antigen-specific CD4 T cells from known pathogen-derived proteins, these approaches are limited for end-stage analyses and do not enable approaches such as T-cell receptor repertoire analyses or gene expression profiling. Single-epitope identification with the linkage of the presenting HLA protein enables multimer-based isolation of CD4 T cells for transcriptional profiling and for tracking the trajectory and diversity of the T-cell repertoire over time in humans (53–55).

The process of epitope discovery described here, assigning both the peptide and the HLA-DR presenting molecule, is distinct from the many studies of SARS-CoV-2 T-cell immunity. First, it is entirely empirical and unbiased in the evaluation of potential candidate epitopes and did not narrow the candidates for study by predictive algorithms (52). Second, epitope discovery was performed *ex vivo* directly. A strategy termed “tetramer-guided epitope mapping “TGEM” (56) has been used successfully to identify peptide:HLA-DR complexes (57), but differs from our two-stage process in several respects. TGEM, like other studies of T-cell responses to SARS-CoV-2 (reviewed in reference 58) or influenza, typically involves an initial *in vitro* expansion of peptide-stimulated or protein-stimulated PBMC for the identification of peptide:HLA-DR complexes, which can shift the representation of peptide specificities. Cytokine ELISpots will only identify cells that produce sufficient cytokines for detection and may poorly detect cells such as Tfh, while tetramers are likely to detect only T cells expressing high-affinity T-cell receptors (59). Despite the differences in assays, we did note several of the same HLA-DR-restricted peptides were identified in both tetramer-based identification and our studies, including a spike peptide presented by HLA-DRB1*15:01 in our studies beginning at amino acid 750 “NLLLQYGSFCTQLNRAL” that is likely the same epitope identified in those by tetramer-guided epitope mapping. Spike epitope (“Spk aa801” NFSQILPDPSKPSKRSFIED), presented by both HLA-DRB1*03:01 and HLA-DRB1*04:01, was discovered in both systems, although this long peptide likely was bound in different registers by these distinctive HLA-DR proteins. The sensitivity of alternative methods for quantification of epitope-specific T cells is difficult to compare because of the alternative methods and peptides used in our studies, tetramer studies, and the AIM assay, used in many assays on SARS-CoV-2 (see references 51, 58, 60 for reviews).

In our two-step epitope discovery process, we did note some differences in the immunodominance of peptides in DR-transgenic mice and HLA-typed human donors. Reactivity in humans may reflect variability in the number and type (e.g., infection and/or vaccination) of confrontations humans have experienced over their lifetime and thus may become enriched for conserved epitopes, while relatively modest in primary responses studied in our first stage of epitope discovery in humanized mice. Also, competition in CD4 T-cell responses among alternate HLA-class II molecules in the typical human host may modulate the relative immunodominance of the particular peptide:HLA-DR tested (61, 62) relative to the single HLA-DR molecule expressed in the mice. The relative immunodominance in mice and humans may also reflect differences in selection of the T-cell receptor repertoire, using mouse TcR genes being selected on human DR. Finally, immune responses in HLA-DR transgenic mice may be modulated by suboptimal interactions between the human HLA-DR protein and the mouse peptide editor DM (H-2M). CD4 T-cell interactions with class II as a “co-receptor” may also be suboptimal in DR-transgenic mice, as only some (HLA-DRB1*01:01 and HLA-DRB1*04:01) incorporated the mouse β2 domain into the HLA-DR transgene, the region of MHC-class II which has been identified as a dominant site for CD4 T-cell interaction (63). Thus, the quantitation and experimental pathway presented here are best used as a positive indicator of the immunogenicity of epitope-specific CD4 T cells that have the potential to be elicited in humans after infection or vaccination. Subjects of different ages, varying histories of infection, and/or vaccination, and different arrays of co-expressed HLA-class II alleles will likely display variability in CD4 T-cell epitope prominence.

There are several limitations in this study. First, some proteins in influenza were not screened for epitopes with HLA-DR transgenic mice because of the pathogenicity of some of the viruses in the HLA-DR transgenic mice. For example, the polymerase protein epitopes were not tested, although we have found that this protein can be immunogenic in several inbred strains of mice (64, 65) and could thus contain highly conserved CD4 T-cell epitopes. Second, we were limited in the targets from SARS-CoV-2 that could be mapped for CD4 T-cell epitopes due to the limited availability of recombinant proteins for vaccination. Third, the relative reactivity in the human samples was drawn from 12 subjects or less per epitope, so the positive response is informative, but the magnitude is less so. We have only studied four HLA-DR alleles, which, although highly represented in the US human population, may have incomplete population coverage, depending on the global population studied. Finally, we did not selectively recruit HLA-typed subjects who have a history of infection, and thus the relative immunodominance among epitopes may shift pre- and post-infection. However, our ability to detect and characterize the epitope-specific CD4 T cells in resting memory cells, without *in vitro* expansion, does demonstrate the fidelity of the approach we have adopted here, providing a framework for future studies of this type. Our studies enable future experiments to gain new insight into the memory pool of influenza and SARS-CoV-2 CD4 T cells as done here, by tetramer analyses or further analyses of T-cell receptor repertoire changes over time with multiple confrontations. Overall, the scope of our studies, involving 4 alleles of HLA-DR, using a two-step screening strategy, and examination of more than eight different pathogen-derived proteins, and testing in 6–12 human subjects for every single peptide using the ELISpot assay has not been described in the literature and provides a framework for the field for future studies.

## MATERIALS AND METHODS

### Mice

The HLA-DR1 (B10.M/J-TgN-DR1) and HLA-DR4 (C57BL/6Tac-Abb < tm > TgNDR4) transgenic mice (66, 67) were obtained from D. Zaller (Merck) through Taconic Laboratories and were maintained in the specific-pathogen-free facility at the University of Rochester according to institutional guidelines. The HLA-DR15 mice (68) were shared by A. Vandenbark (Oregon Health Sciences Center). HLA-DR3 mice (69) on the NOD background were purchased from Jackson Labs (Strain #030434) and backcrossed to the class II null B6 mice developed by Mathis and colleagues (70). Mice were used at 2–8 months of age.

### Proteins and peptides

Influenza peptide arrays are described in Table S1, showing their sequence, length, purity, and source. Peptides were reconstituted at 10 mM in PBS, with or without added dimethyl sulfoxide to increase the solubility of hydrophobic peptides and 1 mM dithiothreitol for cysteine-containing peptides. Overlapping peptide arrays were either pooled together or divided into separate pools for very large proteins. For some proteins, peptides were combined into pools containing 8–12 peptides and arrayed as described previously (33, 71) and illustrated in Fig. 2C and D which enables the identification of immunodominant peptides and exclusion of non-stimulatory peptides. Peptide pools were considered positive if they were at least threefold over the background of no peptides in two experiments (typically greater than 50 spots/million), based on previous experience and other investigators, after which peptides in non-stimulatory pools were eliminated from further study.

### Influenza virus infections and protein vaccination

For infection, mice were anesthetized by intraperitoneal injection with tribromoethanol (Avertin, 14 μL/mg body weight), and virus, diluted to a total volume of 30 μL in PBS, was instilled intranasally. Influenza B/Brisbane/60/2008 and B/Florida/04/2006, provided by Richard Webby, have been previously described (72), A/Hawaii/70/2019 (H1N1) is a non-mouse adapted H1N1 virus, and mouse-adapted A/Switzerland/9,715,293/2013 (H3N2) (73) was kindly provided by F. Krammer, Mt. Sinai, NY. Non-lethal but immunogenic doses were determined for each virus and HLA-DR transgenic strain, typically the range for IBV was 750–1,200 pfu, for H1N1 was 1,000–1,200 pfu, and for H3N2 was 1,200–2,000 pfu. At 10–12 days post-infection, single-cell suspensions were prepared from mediastinal lymph nodes (mLN) and spleen. For protein vaccination, mice were immunized subcutaneously in the rear footpads with 2–5 μg of protein in sterile PBS emulsified in Sigma Adjuvant system (Sigma) or alum (Invivogen). Recombinant SARS-CoV-2 NSP1, NSP5, NSP7/8, NSP9, and NSP15 proteins were cloned in-house (Argonne National Laboratory), SARS-CoV-2 helicase protein was purchased from Sino Biological, and SARS-CoV-2 full-length spike protein (Wuhan Hu-1) and nucleocapsid proteins were produced as described, respectively (74, 75). Ten to twelve days post-vaccination, mice were euthanized, and single-cell suspensions were prepared from the draining popliteal lymph node (pLN) and spleen.

### ELISpot assays

CD4 T cells from secondary lymphoid tissue were enriched by MACS (Miltenyi Biotec) negative selection to deplete CD8 T cells, NK cells, and antigen-presenting cells, following the manufacturer’s recommendation, and subsequently used in ELISpot assays. Assays of purity estimate >90% CD4 T cells with minimal representation of CD8 T cells. The cytokine assays were performed as previously described (6, 37, 76). Briefly, 96-well filter plates (Millipore) were coated with 2 μg/mL purified rat anti-mouse interleukin-2 (IL-2) or IFN-γ (clone JES6-1A12 or AN-18, respectively, BD Biosciences) in PBS overnight at 4°C, washed to remove unbound antibody, and incubated with complete medium containing 10% FBS (Gibco) for 1 h to block nonspecific binding. Purified CD4 T cells from draining lymph nodes (150,000–200,000 cells per well), or spleen (300,000 cells per well) were co-cultured with 500,000 syngeneic splenocytes (HLA-DR4, HLA-DR3, or HLA-DR15). For DR1-Tg mice, which express the mouse I-A^f^ molecule, which poorly selects a CD4 T-cell repertoire, DAP.3 fibroblast cells transfected with the genes encoding HLA-DR1, generously provided by E. Long (NIAID, NIH; 35,000 cells/well) were used, with untransfected DAP.3 cells used as a control. Cultures were incubated with either a pool of peptides or a single peptide at a final concentration of 0.5 μM or 1 μM, respectively, in a total volume of 200 μL for 18 to 20 h at 37°C and 5% CO_2_. After incubation, plates were washed (1× PBS, 0.1% Tween20), and then biotinylated rat anti-mouse IL-2 or IFN-γ (clone JES6-5H or XMG1.2, respectively, BD Biosciences) was added (2 μg/mL in wash buffer with 10% FBS). After incubation and washing, streptavidin-conjugated alkaline phosphatase (Jackson Immuno Research) was added at a dilution of 1:1,000 (1 mg/mL stock) in wash buffer with 10% FBS at 50 μL/well, incubated for 30 min at room temperature, washed, and developed using Vector Blue substrate (Vector Laboratories). Quantification of spots was performed with an Immunospot reader series 5.2. CD4 T cells, APC, and media with no added peptide were used for negative control, and all conditions were performed in duplicate or triplicate. All responses with background subtracted are shown; average values that are at least threefold over background were considered a positive response for further study. Representative ELISpot images are shown in Fig. S1.

### Human ELISpot assay

HLA-typed human cryopreserved PBMC collected between 2018 and 2023 from de-identified healthy subjects aged 19–54, for which no known history of infection or vaccination was recorded, were obtained from C.T.L. (Shaker Hts, OH). After thawing at 37°C and overnight rest in culture, cells were depleted of CD8 and CD56 cells using MACS microbeads per the manufacturer’s instructions (Miltenyi Biotec). ELISpot assays were performed as previously described (45). Briefly, CD8- and CD56-depleted PBMCs (300,000–400,000 cells per well) were cultured with single peptides on plates coated with 10 μg/mL anti-human IFNγ (clone 1-D1K, MabTech) for 36 h at 37°C, 5% CO_2_. After incubation, plates were washed and incubated with biotinylated detection antibody human IFNγ (2 μg/mL, clone 7-B6-1, MabTech) for 2 h, followed by washing and incubation with streptavidin-conjugated alkaline phosphatase (1:1,000 dilution of 1 mg/mL stock) and development using Vector Blue substrate. Data are presented as the frequency of cytokine-producing cells per million CD8- and CD56-depleted PBMCs, to enrich for CD4 T cells and syngeneic antigen-presenting cells. Early experiments indicated that CD8 T-cell reactivity was not detected after negative selection columns. All responses with background subtracted are shown, including zero values. Representative ELISpot images are shown in Fig. S1.

### Human donor HLA typing for tetramer analyses

HLA typing of apheresis de-identified donor samples for tetramer analyses was performed using the AllType NGS 11-Loci Amplification Kit (One Lambda) according to the manufacturer’s instructions. Resulting libraries were sequenced on MiSeq lane at 150 × 150 bp. HLA types were called using the TypeStream Visual Software from One Lambda. Screening was first performed by the ELISpot assay.

### Preparation of U-Load peptide-HLA monomers and tetramer assembly

Unfolded, biotinylated U-Load monomers (Immudex) were obtained for HLA-DRB1*04:01. Influenza virus peptide IAV M1 p17 was commercially synthesized, diluted to 1 mM in DMSO, and loaded into U-Load HLA-DRB1*04:01 according to the manufacturer’s instructions at 37°C for 20 h. An HLA-DRB1*04:01 negative control, containing the CLIP peptide, was also folded. Custom HLA-DRB1*04:01 monomers, loaded with IAV H3p81 or SARS-CoV-2 Sp8, were generated by Immudex. Peptide-HLA tetramers were then assembled according to the manufacturer’s instructions. Briefly, 0.48 ng of fluorochrome-conjugated streptavidin (PE or PE-Cy7, Biolegend) was added to peptide-HLA complex (500 nM in 1× PBS and 5% vol/vol glycerol) in three equal volumes for a total of 60 μL. After each 1/3 volume addition, samples were mixed and incubated for 10 min at 4°C in the dark. Assembled tetramers were stored at 4°C in the dark until use.

### Tetramer-associated magnetic enrichment

PBMCs were isolated from apheresis rings, which are the centrifuge-isolated PBMC populations obtained from the St. Jude Children’s Research Hospital Blood Donor Center under Department of Pathology protocol BDC035. All samples were de-identified before release, and therefore no information is available regarding infection or vaccination history. PBMCs (3–12 × 10^6^) were treated with human TruStain FcX blocking buffer (Biolegend) for 15 min at 4°C in FACS buffer (1× PBS, 0.5% BSA, 0.5 mM EDTA), followed by centrifugation and re-suspension in 50 nM dasatinib (Sigma-Aldrich) in 1× PBS and incubated for 30 min at 37°C and 5% CO_2_. PBMCs were then centrifuged and stained with pairs of PE- and PE/Cy7-conjugated tetramers (1.5 μL tetramer per 1 × 10^6^ PBMCs) for each target (1:10 dilution in FACS buffer containing 500 μM D-biotin) in a volume of 100 μL. PBMCs were incubated with tetramers at 25°C for 1 h with periodic gentle mixing. Tetramer-associated magnetic enrichment (77), using Miltenyi LS columns, was used to select tetramer-bound cells as per the manufacturer’s instructions. Briefly, tetramer-stained PBMCs were washed, and then 20 μL each of anti-PE and anti-Cy7 microbeads (Miltenyi Biotec) was added, cells were gently mixed, and incubated on ice for 30 min. Cells were then washed and re-suspended in MACS buffer before being added to an LS column. The columns were removed from the magnet and washed to collect the tetramer-bound (TAME) fraction. The TAME cell fractions as well as an aliquot of PBMCs not run through the column were pelleted and suspended in FACS buffer containing 500 μM D-biotin (FACS-B) and a cocktail of fluorophore-conjugated surface antibodies (Table S2) in a final volume of 100 μL and incubated in the dark on ice for 30 min. PBMCs were then washed twice and suspended in 250 μL FACS-B. Samples were analyzed on a 5-laser Aurora spectral cytometer. Cell population gating and fluorescence analysis were performed using FlowJo version 10.7.2 software (BD Biosciences). Additional analysis was performed in R (v4.1.0).

## Data Availability

The full complement of data accumulated for these studies is available upon request.

## SUPPLEMENTAL MATERIAL

Supplemental Tables and Figure mbio.00449-26-s0001.pdf Table S1 to S5; Figure S1.

ASM does not own the copyrights to Supplemental Material that may be linked to, or accessed through, an article. The authors have granted ASM a non-exclusive, world-wide license to publish the Supplemental Material files. Please contact the corresponding author directly for reuse.
