# Supplementary material for: A funnel approach to enable analyses of epitope-specific human CD4 T cells specific for influenza and SARS-CoV-2
Source: mBio. 2026 May 19;17(6):e00449-26. doi: 10.1128/mbio.00449-26 (PMC13251400; doi:10.1128/mbio.00449-26)
Supplement: Supplemental Tables and Figure — Table S1 to S5; Figure S1. [file mbio.00449-26-s0001.pdf]

	Protein	Number of Peptides	Length of peptide	Amino Acid Overlap	Purity Range	Source	BEI Resources (BEIR) Reference Number
H1N1 A/California/04/2009	HA	139	15	11	80-99%	BEIR	NR-15433
	NA	115	13-16	10-12	80-99%	BEIR	NR-18975
	NP	122	14-15	11	80-99%	BEIR	NR-18976
	M1	61	11-16	10-12	80-99%	BEIR	NR-18977
	NS1	52	14-17	10-13	80-99%	BEIR	NR-18979
H1N1 A/New Caledonia/20/1999	HA	94	17	11	65-85%	BEIR	NR-2602
	NA	78	17	11	70-98%	BEIR	NR-2606
H1N1 A/New York/348/2003	NP	82	17	11	80-98%	BEIR	NR-2611
	M1	41	17	11	80-99%	BEIR	NR-2613
H3N2 A/Perth/16/2009	HA	139	15	11	82-99%	BEIR	NR-19266
IBV B/Brisbane/60/2008	HA	144	13-15	11	85-99%	BEIR	NR-19247
	NA	114	14-15	11	80-99%	BEIR	NR-19253
IBV B/Florida/04/2006	HA	96	17	11	80-99%	BEIR	NR-18972
	NA	114	13-16	10-12	70-99%	BEIR	NR-19254
	NP	110	15-17	11	65-85%	BEIR	NR-36045
	M1	47	17	10-12	72-99%	BEIR	NR-36046
	NS1	68	16	11	70-85%	Mimotopes	
SARS-CoV-2	NCP	59	17 or 13	10	80-95%	BEIR	NR-52404
	Spike	253	17	11	70-95%	Mimotopes	
	Helicase	119	17	11	70-95%	Mimotopes	
	NSP1	36	17	11	70-95%	Mimotopes	
	NSP5	61	17	11	70-95%	Mimotopes	
	NSP7	16	17	11	70-95%	Mimotopes	
	NSP8	39	17	11	70-95%	Mimotopes	
	NSP9	21	17	11	70-95%	Mimotopes	
	NSP15	67	17	11	70-95%	Mimotopes	

Supplemental Table I. Detailed information for peptide arrays.

Antibody	Fluorochrome	Clone	Dilution	Supplier	Item Identifier
CD95	BUV395	DX2	1:20	BD Biosciences	740306
CD62L	BUV496	SK11	1:20	BD Biosciences	750589
CD28	BUV563	CD28.2	1:20	BD Biosciences	741392
CD45RA	BV421	HI100	1:20	Biolegend	304130
CD25	V450	M-A251	1:20	BD Biosciences	560355
CD45RO	BV605	UCHL1	1:20	Biolegend	304238
CD4	BV650	SK3	1:20	Biolegend	344692
CD11a	BV711	HI111	1:20	Biolegend	301238
CD27	BV785	O323	1:20	Biolegend	302832
CCR7	FITC	G043H7	1:20	Biolegend	353216
CD58	PerCP-Cy5.5	TS2/9	1:20	Biolegend	330914
CD3	PerCP-eFluor 710	OKT3	1:20	ThermoFisher	46-0037-42
NKG2D	APC	1D11	1:20	Biolegend	320808
CXCR3	AF700	G025H7	1:20	Biolegend	353742
Live/Dead	BV510		1:400	Tonbo	13-0870-T500

Supplemental Table II. Antibodies used in tetramer staining panel.

HLA	Subject ID	Collection Date	Age at Draw	Sex	Ethnicity	Symbol	Color
DRB1*01:01	268	5/2017	21	M	Hispanic	□	red
	299	6/2018	28	F	Hispanic/Latino		yellow
	651	8/2023	33	M	Caucasian		purple
	652	8/2023	34	M	Caucasian		pink
	659	9/2023	54	F	Caucasian		blue
	618	3/2023	49	M	Caucasian		green
	664	10/2023	24	F	Caucasian		turquoise
	594	5/2023	25	M	Caucasian		orange
	605	9/2022	42	F	Caucasian		dark blue
	629	4/2023	46	F	Caucasian		black
DRB1*04:01	572	3/2022	24	F	Caucasian	○	red
	578	3/2022	40	M	Caucasian		yellow
	587	4/2022	50	M	Caucasian		green
	698	6/2022	38	F	Caucasian		blue
	643	7/2023	38	M	Caucasian		purple
	603	9/2022	31	F	Caucasian		turquoise
	597	6/2022	38	F	Caucasian		pink
	577	3/2022	41	F	African/American		orange
	670	11/2023	19	F	Caucasian		grey
	493	4/2021	49	F	Caucasian		white
	531	9/2021	43	F	Caucasian		dark blue
	500	5/2021	19	M	Caucasian		black
DRB1*03:01	521	7/2021	20	M	Caucasian	▽	pink
	522	8/2021	37	F	Native American		turquoise
	533	9/2021	34	M	Caucasian		white
	568	6/2023	35	M	Caucasian		red
	608	10/2022	42	M	Hispanic		orange
	612	2/2023	21	M	African/American		yellow
	628	4/2023	31	M	Caucasian		green
	660	9/2023	47	F	Caucasian		blue
	673	12/2023	32	F	Caucasian		purple
DRB1*015:01	505	5/2021	32	M	Caucasian	◇	pink
	513	7/2021	26	F	Caucasian		turquoise
	518	7/2021	24	M	Caucasian		white
	655	8/2023	27	M	Caucasian		red
	658	9/2023	27	M	Caucasian		orange
	662	9/2023	21	F	Caucasian		yellow
	668	11/2023	23	M	Caucasian		green
	671	11/2023	51	F	Caucasian		blue
	672	12/2023	42	M	Caucasian		purple

Supplemental Table III. Demographics information and unique symbol identifiers for PBMC samples from healthy adult subjects that were purchased from CTL and used in Figure 10.

Selected HLA	Subject ID	HLA-A	HLA-A	HLA-B	HLA-B	HLA-C	HLA-C	**HLA-DRB1**	HLA-DRB1	HLA-DQB1	HLA-DQB1	HLA-DPB1	HLA-DPB1	HLA-DQA1	HLA-DQA1	HLA-DRB3/4/5	HLA-DRB3/4/5	HLA-DPA1	HLA-DPA1
DRB1*01:01	268	*02:33	*03:01	*14:02	*51:01	*03:04	*08:02	***01:01**	*16:02	*03:01	*05:01	*04:02G	*09:01G			DRB5*02:02	~	*01:03	*02:01
	299	*03:01	*24:02	*08:01	*35:01	*04:01	*07:02	***01:01**	*11:04	*03:01	*05:01	*04:01G	*04:02G			DRB3*02:02	~	*01:03	*01:03
	651	*03:01	*31:01	*15:01	*44:03	*01:02	*16:01	***01:01**	*04:07	*03:02	*05:01	*04:01	*04:01	*01:01	*03:01	DRB4*01:03	~	*01:03	*01:03
	652	*02:01	*02:01	*15:01	*56:01	*01:02	*04:01	***01:01**	*05:01	*05:01	*06:03	*04:01	*05:01	*01:01	*01:03	DRB3*02:02	~	*01:03	*01:03
	659	*02:01	*32:01	*08:01	*14:01	*07:01	*08:02	***01:01**	*07:01	*02:02	*05:01	*04:01	*04:02	*01:01	*02:01	~	DRB4*01:01	*01:03	*01:03
	618	*01:01	*32:01	*37:01	*44:02	*05:01	*06:02	***01:01**	*15:01	*05:01	*06:02	*03:01	*04:01	*01:01	*01:02	DRB5*01:01	~	*01:03	~
	664	*02:01	*02:01	*08:01	*51:01	*03:03	*07:01	***01:01**	*03:01	*02:01	*05:01	*02:01	*03:01	*01:01	*05:01	DRB3*01:01	~	*01:03	*01:03
	594	*03:01	~	*13:02	~	*06:02	~	***01:01**	*07:01	*02:01	*05:01	*02:01	~	*02:01	*01:01	DRB4*01:01	~	*01:03	~
	605	*03:01	*26:01	*07:02	*38:01	*07:02	*12:03	***01:01**	*07:01	*03:03	*05:01	*04:01	*06:01	*01:01	*02:01	DRB4*01:01	~	*01:03	~
	629	*02:01	*11:01	*35:01	*49:01	*04:01	*07:01	***01:01**	*04:02	*03:02	*05:01	*02:01	*04:02	*01:01	*03:01	~	DRB4*01:03	*01:03	*01:03
DRB1*04:01	572	*02:01	*23:01	*44:02	*52:01	*05:01	*12:02	***04:01**	*15:02	*03:01	*06:01	*04:01	*04:02	*01:03	*03:01	DRB4*01:01	DRB5*01:02	*01:03	~
	578	*11:01	*29:02	*35:01	*35:01	*04:01	*16:01	***04:01**	*07:01	*03:01	*03:03	*04:01	*17:01	*02:01	*03:01	DRB4*01:01	~	*01:03	*02:01
	587	*01:01	*24:02	*15:01	*40:01	*03:03	*03:04	***04:01**	*13:02	*03:02	*06:04	*04:01	*14:01	*01:02	*03:01	DRB3*03:01	DRB4*01:01	not tested	not tested
	698	*02:01	*24:02	*07:02	*15:01	*03:03	*07:02	***04:01**	*04:04	*03:02	~	*04:01	*05:01	*03:01	~	DRB4*01:01	~	*01:03	*02:02
	643	*23:01	*31:01	*44:02	*44:03	*05:01	*06:01	***04:01**	*07:01	*02:02	*03:01	*04:01	*11:01	*02:01	*03:03	DRB4*04:01	DRB4*01:03	*01:03	*01:03
	603	*02:01	*26:01	*07:02	*44:02	*05:01	*07:02	***04:01**	*16:01	*03:01	*05:02	*04:01	~	*01:02	*03:01	DRB4*01:01	DRB5*02:02	*01:03	~
	597	*02:01	*24:02	*07:02	*15:01	*03:03	*07:02	***04:01**	*04:04	*03:02	~	*04:01	*05:01	*03:01	~	DRB4*01:01	~	*01:03	*02:02
	577	*02:01	*03:01	*44:02	*49:01	*05:01	*07:01	***04:01**	*01:02	*03:01	*05:01	*02:01	*04:02	*01:01	*03:01	DRB4*01:01	~	*01:03	~
	670	*02:01	*02:01	*07:02	*27:05	*02:02	*07:02	***04:01**	*15:01	*03:01	*06:02	*02:01	*04:01	*01:02	*03:03	DRB4*01:03	DRB5*01:01	*01:03	*01:03
	493	*01:01	*31:01	*27:05	*37:01	*02:02	*06:02	***04:01**	*10:01	*03:02	*05:01	*04:01	*04:02	*01:01	*03:01	DRB4*01:01	~	*01:03	~
	531	*01:01	*68:02	*14:02	*44:02	*06:02	*08:02	***04:01**	*13:03	*03:01	~	*04:01	*11:01	not tested	not tested	DRB3*01:01	DRB4*01:01	not tested	not tested
	500	*02:01	*29:02	*40:01	*44:03	*03:04	*16:01	***04:01**	~	*03:01	*03:02	*02:01	*04:01	*03:01	~	DRB4*01:01	~	*01:03	~
DRB1*03:01	521	*01:01	*25:01	*08:01	*18:01	*07:01	*12:03	***03:01**	*11:01	*02:01	*03:01	*04:01	~	*05:01	~	DRB3*01:01	DRB3*02:02	*01:03	~
	522	*01:01	*02:01	*08:01	*15:01	*03:04	*07:01	***03:01**	*04:04	*02:01	*03:02	*01:01	*06:01	*03:01	*05:01	DRB3*01:01	DRB4*01:01	*01:03	*02:01
	533	*01:01	*02:01	*08:01	*15:18	*07:01	*07:04	***03:01**	*15:02	*02:01	*06:01	*04:01	*04:02	not tested	not tested	DRB3*01:01	DRB5*01:02	not tested	not tested
	568	*01:01	*02:01	*07:02	*08:01	*07:01	*07:02	***03:01**	*09:01	*02:01	*03:03	*01:01	*14:01	*03:01	*05:01	DRB3*01:01	DRB4*01:01	*02:01	*02:01
	608	*01:01	*33:03	*51:01	*53:01	*04:01	*14:02	***03:01**	*07:01	*02:01	~	*04:02	~	*02:01	*05:01	DRB3*02:02	DRB4*01:01	*01:03	~
	612	*29:02	*30:02	*08:01	*49:01	*07:01	*07:01	***03:01**	*15:03	*02:01	*06:02	*105:01	*665:01	*01:02	*05:01	DRB3*02:02	DRB5*01:01	*03:01	*04:02
	628	*01:01	*02:01	*08:01	*08:01	*07:01	*07:01	***03:01**	*03:01	*02:01	*02:01	*01:01	*01:01	*05:01	*05:01	DRB3*01:01	DRB3*01:01	*02:01	*02:01
	660	*02:01	*03:01	*08:01	*51:01	*01:02	*07:01	***03:01**	*11:01	*02:01	*03:01	*04:01	*04:01	*05:01	*05:05	DRB3*01:01	DRB3*02:02	*01:03	*01:03
	673	*01:01	*30:02	*08:01	*18:01	*05:01	*07:01	***03:01**	*03:01	*02:01	*02:01	*02:01	*04:01	*05:01	*05:01	DRB3*01:01	DRB3*02:02	*01:03	*01:03
DRB1*15:01	505	*02:01	*32:01	*27:05	*44:02	*01:02	*05:01	***15:01**	*11:76	*03:01	*06:02	*02:02	*04:01	*01:02	*05:01	DRB3*02:02	DRB5*01:01	*01:03	~
	513	*02:01	~	*07:02	*18:01	*07:02	*07:04	***15:01**	*11:01	*06:02	~	*02:01	*04:01	*01:02	~	DRB3*02:02	DRB5*01:01	*01:03	~
	518	*03:01	*24:02	*40:01	*40:02	*02:02	*03:04	***15:01**	*08:01	*04:02	*06:02	*04:02	~	*01:02	*04:01	DRB5*01:01	~	*01:03	~
	655	*03:01	*03:01	*12:02	*51:01	*01:02	*06:02	***15:01**	*07:01	*02:02	*06:03	*02:01	*15:01	*01:02	*02:01	DRB4*01:03	DRB5*01:01	*01:03	*01:03
	658	*03:01	*26:01	*07:02	*08:01	*07:01	*07:02	***15:01**	*11:11	*03:01	*06:02	*04:01	*04:01	*01:02	*05:05	DRB3*02:02	DRB5*01:01	*01:03	*01:03
	662	*02:01	*33:03	*15:16	*18:01	*12:03	*14:02	***15:01**	*01:02	*05:01	*06:02	*13:01	*23:01	*01:01	*01:02	~	DRB5*01:01	*01:03	*02:01
	668	*02:01	*03:01	*39:06	*51:01	*01:02	*07:02	***15:01**	*11:01	*03:01	*06:02	*01:01	*02:02	*01:02	*05:05	DRB3*02:02	DRB5*01:01	*01:03	*02:01
	671	*01:01	*23:17	*35:01	*27:01	*06:02	*07:19	***15:01**	*07:01	*03:03	*06:02	*04:01	*104:01	*01:02	*02:01	DRB4*01:03	DRB5*01:01	*01:03	*01:03
	672	*01:01	*24:02	*07:02	*08:01	*07:01	*07:02	***15:01**	*15:01	*06:02	*06:02	*04:01	*04:01	*01:02	*12:02	DRB5*01:01	DRB5*01:01	*01:03	*01:03

Supplemental Table IV. Full HLA Report for PBMC samples used in Elispot assays.

**ID**	**A_1**	**A_2**	**B_1**	**B_2**	**C_1**	**C_2**	**DPA1_1**	**DPA1_2**	**DPB1_1**	**DPB1_2**	**DQA1_1**	**DQA1_2**	**DQB1_1**	**DQB1_2**	**DRB1_1**	**DRB1_2**	**DRB345_1**	**DRB345_2**
Subject 17	*03:01	*26:01	*07:02	*15:18	*07:02	*07:04	*01:03	*01:03	*04:01	*04:01	*03:03	*03:03	*03:01	*03:01	***04:01**	***04:01**	DRB4*01:03	DRB4*01:03
Subject 31	*02:01:01:01	*32:01:01:01	*38:01:01:01	*44:02:01:01	*05:01:01	*12:03:01:01	*01:03:01	*01:03:01	*02:01:02	*02:01:02	*03:03:01:01	*05:05:01:02	*03:01:01:01	*03:01:01:03	***04:01:01:01**	*11:04:01	DRB3*02:02:01:02	DRB4*01:03:01
Subject 38	*03:01:01:01	*26:01:01:01	*07:02:01:01	*15:18:01:05	*07:02:01:03	*07:04:01	*01:03:01:04	*01:03:01:04	*04:01:01	*04:01:01	*03:03:01:01	*03:03:01	*03:01	*03:01:01:01	***04:01:01**	***04:01:01:01**	DRB4*01:03:01	DRB4*01:03:01
Subject 39	*24:02	*24:02	*15:01:01:01	*51:01:01	*01:02:01:01	*03:03:01	*01:03:01	*02:02:05	*04:01:01	*19:01:01	*01:03:01:02	*03:03	*03:02	*06:03:01:01	***04:01:01**	*13:01:01	DRB3*02:02:01:02	DRB4*01:03:01
Subject 40	*03:01:01:01	*26:01:01:01	*07:02:01:01	*15:18:01:05	*07:02:01:03	*07:04:01	*01:03:01:04	*01:03:01:04	*04:01:01	*04:01:01	*03:03:01:01	*03:03:01	*03:01	*03:01:01:01	***04:01:01**	***04:01:01:01**	DRB4*01:03:01	DRB4*01:03:01
Subject 52	*26:01:01:01	*68:01:01:02	*27:05:02:05	*35:03:01:03	*01:02:01:01	*04:01:01	*01:03:01	*01:03:01:20	*04:01:01	*04:02:0	*01:01:01:01	*03:03:01	*03:01:01:01	*05:01:01:03	*01:01:01	***04:01:01**	DRB4*01:03:01	DRB4*01:03:01

Supplemental Table V. Full HLA Report for PBMC samples used in tetramer studies.

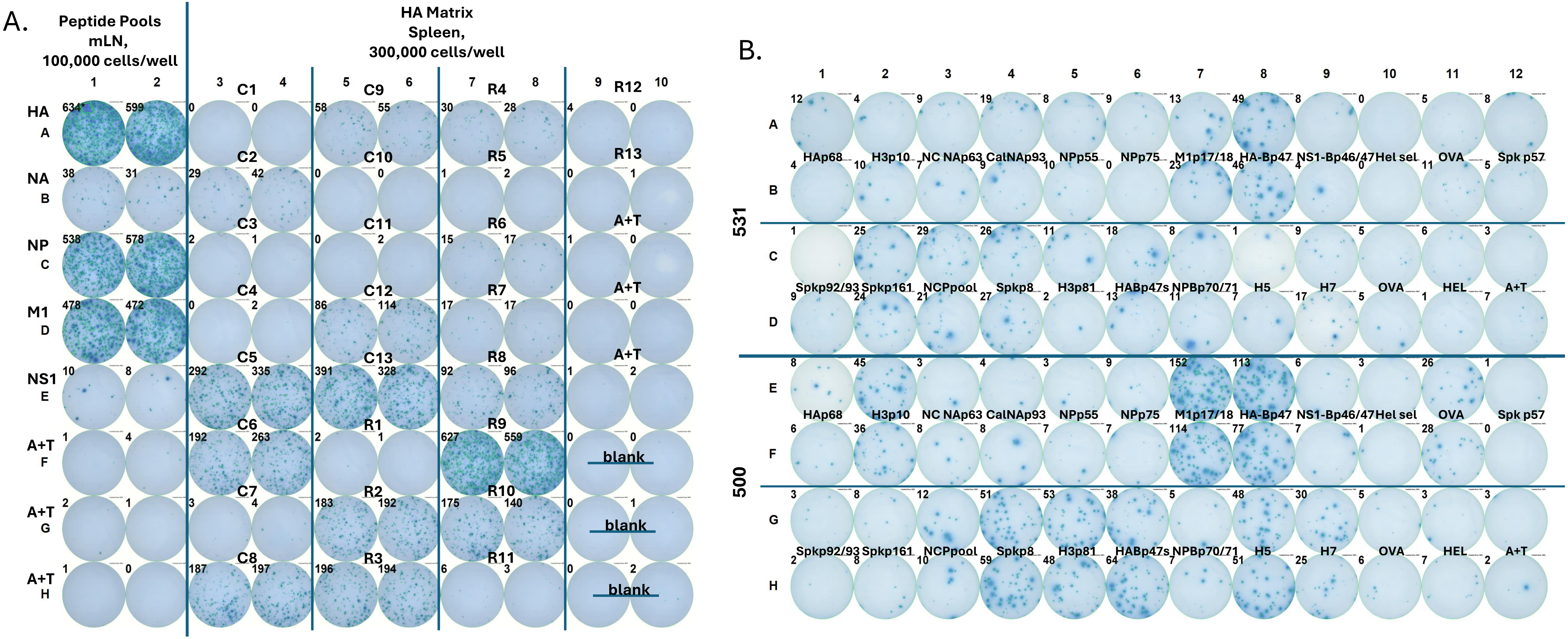

Supplemental Figure 1. Representative ELISpot images from mouse mLN and spleen (A) and human PBMC (B) IFNγ assays. Wells are labels with the pool of peptides as described in Methods and Figure 2A and B, or the single peptides, as shown in Figure 10, used for stimulation.
